# Translational advances in diabetic nephropathy management: integrating nanoformulation-based drug delivery systems

**DOI:** 10.3389/fmedt.2026.1951734

**Published:** 2026-08-25

**Authors:** Mohd Danish Ansari, Ashish Vishwakarma, Md Quamuddin, Mansi Aggarwal, Hashim Khan, Muhammad Fauzi Abd Jalil, Syed Mahmood, Adil Farooq Wali, Sirajunisa Talath, Mohamed El-Tanani

**Affiliations:** 1 Department of Pharmaceutical Technology, Faculty of Pharmacy, Universiti Malaya, Kuala Lumpur, Malaysia; 2 Department of Pharmacognosy & Phytochemistry, School of Pharmaceutical Sciences, Delhi Pharmaceutical Sciences and Research University, New Delhi, India; 3 Metro College of Health Sciences and Research, Greater Noida, India; 4 Raj Kumar Goel Institute of Technology (Pharmacy), Ghaziabad, India; 5 RAK College of Pharmacy, RAK Medical and Health Sciences University, Ras Al Khaimah, United Arab Emirates

**Keywords:** antifibrotic therapy, chronic kidney disease, diabetic nephropathy, nanomedicine, nanotechnology, renal targeting

## Abstract

The incidence of diabetes mellitus (DM) is increasing worldwide, especially for its major renal complications, diabetic nephropathy (DN) and chronic kidney disease (CKD). These are caused by chronic hyperglycemia, oxidative stress, hemodynamics, inflammation, and profibrotic signaling, which result in progressive renal structural damage and functional decline. All these mechanisms contribute to podocyte injury, thickening of the glomerular basement membrane, interstitial fibrosis, and progressive loss of renal function. While traditional diagnostic markers like albuminuria or estimated glomerular filtration rate (eGFR) aid in tracking disease with imaging, and emerging markers like neutrophil gelatinase-associated lipocalin (NGAL), kidney injury molecule-1 (KIM-1), and cystatin C can help, none can detect early, subclinical kidney damage. A comprehensive literature review was conducted in PubMed, Google Scholar, Scopus, Web of Science, and ScienceDirect using keywords related to DN pathophysiology, diagnosis, nanomedicine, renal targeting, and nanoformulations, with publications between 2015 and 2026. Intensive glycemic and blood pressure control, inhibition of the renin-angiotensin-aldosterone system, and lipid-lowering therapy are currently used as therapeutic interventions. Poor bioavailability, off-target toxicity, complex dosing regimens, and variable renal drug handling are often responsible for their limited effectiveness. One approach to circumventing these limitations is with nanotechnology-based drug delivery systems. In preclinical studies, the antioxidant, anti-inflammatory, and antifibrotic effects have consistently been reported to be superior, with reduced systemic toxicity, while clinical translation remains in its early stages. This review discusses current knowledge on DN and critically evaluates the pros, cons, and future of nanotechnology-based approaches for delivering targeted nanomedicines to the kidney.

## 1 Introduction

DM is a chronic metabolic disorder characterized by elevated blood glucose levels due to defects in insulin secretion, action, or both. It disrupts glucose, lipid, and protein metabolism. DM is one of the most urgent worldwide health concerns of the 21st century. Diabetes affected 828 million people globally in 2022, almost four times as many as in 1990, with the greatest increase in low- and middle-income countries (1). Projections suggest that the global diabetic population may exceed 783 million by 2045, driven by obesity, sedentary lifestyles, and aging demographics. In addition to its increasing incidence, diabetes is linked to several crippling complications, such as heart disease, stroke, retinopathy, neuropathy, nephropathy, diabetic foot ulcers, and heightened vulnerability to infections such as tuberculosis and severe COVID-19 (2, 3). These complications collectively contribute to a significant reduction in quality of life, high disability-adjusted life years (DALYs), and premature mortality, underscoring the urgent need for effective preventive and therapeutic strategies (4, 5).

Among these problems, DN is one of the most serious and common, which affects about one-third of diabetic patients. DN is the major cause of CKD and ESRD globally, accounting for up to 50% of ESRD cases requiring dialysis or transplantation (6, 7). Clinically, DN is characterized by persistent albuminuria, progressive fall in glomerular filtration rate (GFR), renal fibrosis, and final renal failure. Progression of renal pathology not only leads to renal morbidity but also greatly raises the risk of cardiovascular disease, thereby presenting a double jeopardy for life (8, 9).

DN is a multifactorial and complex disease.

Chronic hyperglycemia places hemodynamic stress and metabolic dysfunction on the kidney, leading to glomerular hyperfiltration, mesangial expansion, podocyte injury, and thickening of the glomerular basement membrane (10, 11). Pathways that mediate oxidative stress, inflammation, advanced glycation end products (AGEs), the renin-angiotensin–aldosterone system (RAAS), transforming growth factor-β (TGF-β), and NF-κB signaling all contribute to fibrosis and renal dysfunction. Although therapeutic management has improved with the use of insulin and oral antidiabetic drugs (OADs), renoprotective drugs such as ACE inhibitors and ARBs, and newer classes such as SGLT2 inhibitors and GLP-1 analogs, there is still no complete cure for DN. Current treatment strategies are further restricted by the limitations of poor drug solubility, low bioavailability, off-target toxicity, and poor renal targeting (12–16).

A possible avenue in light of these challenges is the use of nanotechnology-based therapies. Nanocarriers, such as liposomes, solid lipid nanoparticles, polymeric nanoparticles, dendrimers, micelles, and nanogels, offer multiple advantages: enhanced solubility and stability of therapeutic agents, improved bioavailability, controlled and sustained release, and renal-specific targeting that minimizes systemic toxicity. Furthermore, the development of nanotheranostic platforms enables simultaneous diagnosis and treatment, potentially revolutionizing disease monitoring and management (17–19). While earlier reviews have mainly focused on general information on the applications of nanomedicine in renal disease, this review aims to critically analyze renal targeting strategies and their translational potential. A special focus is given to comparing passive and active targeting strategies, identifying specific intrarenal target areas, such as the glomerular, podocyte, tubular, and mesangial compartments, and analyzing physicochemical properties that influence cellular uptake. Furthermore, this review explores the potential for preclinical results to translate into clinical success, as well as the manufacturing, scalability, reproducibility, safety, and regulatory hurdles along the way. Finally, some nanoformulations with relatively strong translational support are emphasized to identify those with the greatest potential for future human applications, rather than those with only limited support from preliminary preclinical trials.

## 2 Pathophysiology of DN

**Figure 1:**
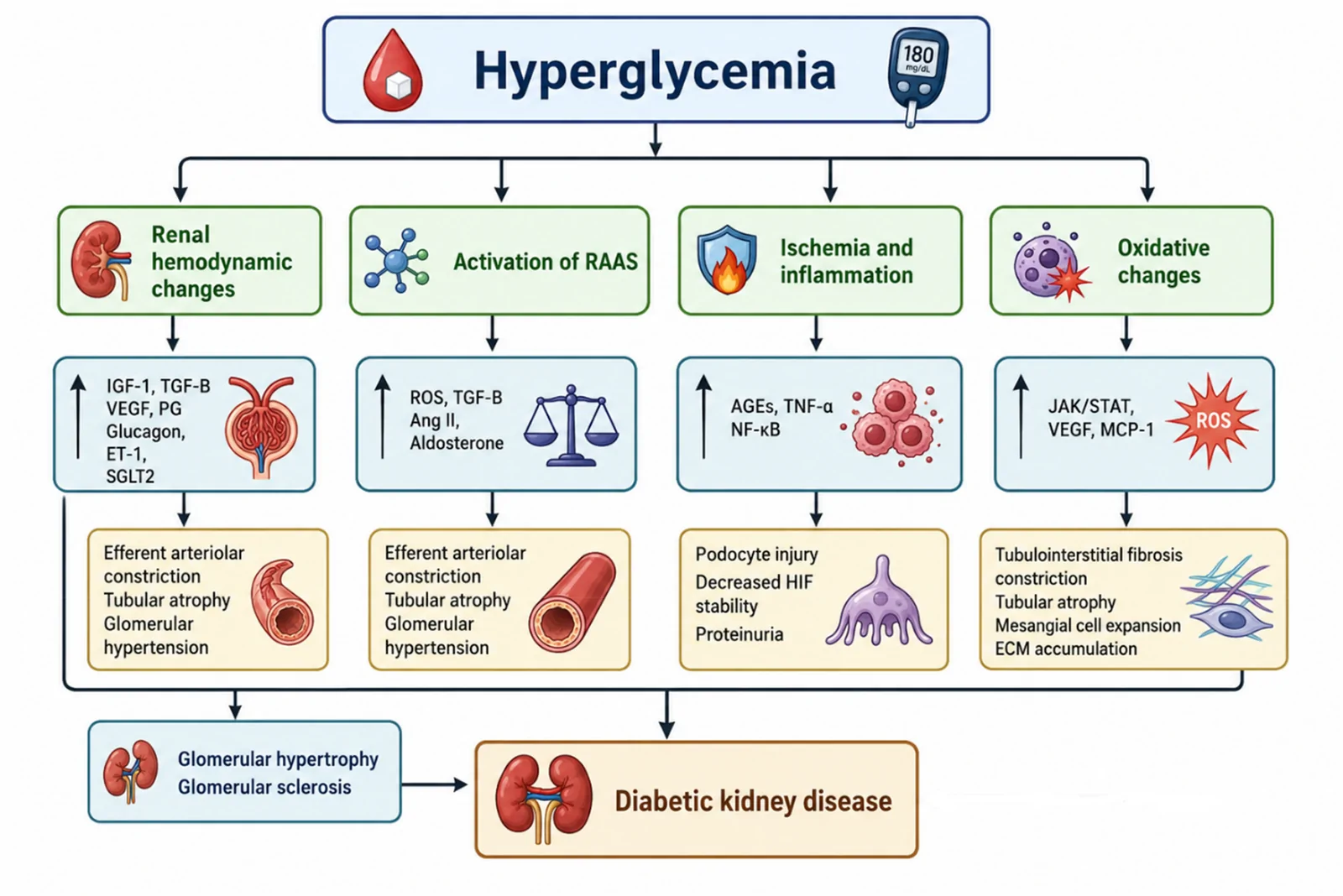
Pathophysiological mechanisms involved in DN.

Pathophysiology of DN in people with DM: kidney function slowly decreases over time (also called DN or DMN). Throughout the world, it is the main cause of ESRD and CKD (20). The pathogenesis of DN is complex and involves metabolic, hemodynamic, growth, and inflammatory variables, and leads to progressive kidney damage as shown in (Figure 1) (21).

### 2.1 Hyperglycemia and metabolic dysfunction

Hyperglycemia is the primary metabolic defect underlying DN and is closely linked to oxidative stress, mitochondrial dysfunction, inflammation, and fibrosis, ultimately leading to increased renal injury via the accumulation of AGEs, mitochondrial DNA damage, and oxidative imbalance. It has been discovered that these disturbances can persist even after glucose levels are brought to normal, over a longer period of time, a phenomenon termed metabolic memory (20). Chronic hyperglycemia alters normal cellular metabolism, leading to increased production of reactive oxygen species (ROS) and dysfunction of mitochondrial activity (21). This metabolic inefficiency amplifies the oxidative stress and perpetuates the vicious cycle between hyperglycemia and oxidative damage in DN.

### 2.2 Role of oxidative stress and inflammation

Oxidative stress is one of the foremost pathogenic mechanisms in DN pathology, causing direct cellular damage and indirectly activating signaling cascades (20, 21).

Prolonged hyperglycemia results in excessive ROS production, including the hydroxyl radical, NO, superoxide anion, hydrogen peroxide, and peroxynitrite. These ROS are generated by mitochondrial overload, the activation of nicotinamide adenine dinucleotide phosphate (NADPH) oxidase (NOX), and AGEs. ROS Cause Direct Damage to DNA, Proteins, and Lipids in renal cells, affecting their structure and functions (20, 22).

Oxidative stress also induces endocytic trafficking perturbations that are related to changes in the mixing of the slit diaphragm. This can then lead to downregulation of slit diaphragm protein production, including podocin and nephrin in podocytes, upregulation of hypertrophy and apoptosis, and foot process effacement. These alterations contribute to the destruction of the Glomerular filtration barrier (GFB) and proteinuria, which are early symptoms of DN. In addition, oxidative stress could induce inflammation through the NF-κB-dependent signaling pathway (23). Indeed, inflammation is also known to play a role in type 1 and type 2 diabetes.

Although studies are still underway to elucidate the causes of diabetes and inflammation, ROS is one of the leading candidates (24, 25). At the same time, ROS are redox-sensitive signaling molecules and indirectly activate various pathways, thereby increasing kidney damage. These pathways involve the induction of proinflammatory cytokines, that is, Tumor Necrosis Factor-alpha (TNF- α), IL-1β, IL-6, and activation of MCP κB signaling (25).

Following infection or stress, the kidney's innate immune cells may form multiprotein complexes, such as NLRP3 (NOD-like receptor protein 3, LRR and pyrin domain-containing protein 3) inflammasomes. Therefore, caspase-1 is activated, promoting inflammation, cell death, and cytokine maturation. Inflammatory responses and the generation of proinflammatory cytokines, such as IL-1β and IL-18, were reported in (21, 22). Additionally, it exacerbates sterile inflammation and renal function decline in DN by promoting gasdermin D (GSDMD) cleavage, leading to pyroptosis characterized by podocyte swelling, membrane pore formation, and IL-1β/IL-18 production. The NLRP3–ASC–caspase-1–IL-1β–IL-18 axis is now recognized to play an important role in the pathogenesis of renal disease.

The conflicting role of macrophages in injury and repair remains to be elucidated, but it may be feasible to dampen inflammation and fibrosis while simultaneously enhancing the kidney's ability to recover by manipulating macrophage behavior or selectively blocking NLRP3 inflammasomes targeting (21).

### 2.3 Podocyte injury and glomerular basement membrane thickening

Podocyte damage is an important factor in the initiation and development of DN (26, 27). Podocytes are a type of epithelial cell that is vital for glomerular integrity and function, enveloping the glomerular capillaries and producing foot processes that comprise the filtration barrier (28). In DN, severe podocyte damage may result from impaired podocyte autophagy, thereby leading to high levels of proteinuria. The presence of proteinuria is significantly associated with a reduced number and compromised structure or function of podocytes, which play a crucial role in maintaining the GFB (27, 29). Elevated glucose concentrations can stimulate the creation of ROS and trigger podocyte apoptosis, resulting in podocyte damage and aggravating DN (28, 30). Consequently, this may result in a reduction of podocyte density, hypertrophy, degeneration, and the effacement of foot processes (31).

One of the first histological evidences of DN is thickening of the glomerular basement membrane (GBM). This mesangial expansion, together with this thickening, leads to glomerular hypertrophy. GBM thickening may be observed 1.5 years after the onset of T1DM in patients with pre-existing disease, even before clinical albuminuria is evident (27, 32). Normal GBM is composed of type-IV collagen (mostly, alpha 3, 4, and 5), laminin, fibronectin, entactin, and proteoglycans. The DN can exhibit increased expression and accumulation of type IV collagen 3 and 4, and a decline in heparan sulfate proteoglycans. Hyperglycemia directly increases the expression of specific collagen mRNAs and proteins in mouse podocytes, thereby contributing to GBM thickening. Other mediators, such as Angiotensin II (Ang II) and Transforming Growth Factor-β (TGF-β), also influence matrix production (27). Growth of the extracellular matrix (ECM), with an imbalance between synthesis and degradation due to hyperglycemia, also contributes to the thickening of the GBM. This thickening is another early indicator of DN and may be predictive of those diabetic individuals who develop progressive nephropathy (32).

### 2.4 Key signaling pathways

The pathogenesis and progression of DN are essential to the role of various signaling pathways. TGF-B is activated by oxidative stress and ROS, and plays a role in most of its pro-fibrogenic activity (20, 33). These outcomes include fibroblast activation, epithelial/endothelial apoptosis, epithelial–mesenchymal transition (EMT), and the production of pro-fibrogenic mediators, including plasminogen activator inhibitor 1 (PAI-1), which eventually lead to fibrosis, decreasing elasticity, increasing the detachment rate of podocytes, and contributing to glomerulosclerosis (20, 27).

The TGF-β/SMAD (Mothers Against Decapentaplegic) signaling pathway is very important in fibrogenesis, particularly in renal fibrosis in DN (34). TGF-B1 triggered an adhesive protein and connexin loss downstream signaling cascade in a high glucose environment. Regarding the pathogenesis of renal fibrosis, SMAD3 is considered pathogenic, while SMAD2 and SMAD7 are protective. TGF- B signaling also increases the expression of pathogenic microRNAs (e.g., miR-21, miR-192, miR-377) and long noncoding RNAs, and reduces the expression of protective microRNAs (e.g., miR-29a/b, miR-200a) to mediate DN. As a means of DN improvement, it has been shown that inhibiting TGF-2 signaling by targeting the ligand, receptor (TGFBR2), SMAD3, downstream microRNAs/lncRNAs, or by overexpressing SMAD7 can be successful (35).

NF- kB pathway is a primary inflammatory mediator in DN (36). NF-KB increases pro-inflammatory cytokines TNF-α, IL-1α, IL-6, and enzymes (COX-2, iNOS) in diabetic kidneys during activation by ROS, AGEs, PKC, and the RAAS (37). Interactions with TGF-b/SMAD signaling also enhance inflammation and fibrosis in the kidney, with SMAD7 serving as the regulatory brake. NF-κB and TGF-β1/SMAD3 activity have been inhibited by renoprotective agents such as wogonin (38). Other signaling pathways, such as JAK/STAT, NLRP3, Wnt/β-catenin, PI3K/Akt, and glucose-dependent pathways (PKC, AGEs, polyol, hexosamine) and vascular endothelial growth factor (VEGF), are also involved in inflammation, immune cell infiltration, and glomerulosclerosis in DN (39, 40).

### 2.5 Biomarkers involved in early and progressive DN

The most widely accepted clinical indicator of DN is microalbuminuria (MA), regarded as the first and most frequently used indicator of the disease (41, 42). Its sensitivity and specificity for forecasting the DN progression are, however, limited (37, 41). Already, patients who show microalbuminuria might have progressed to develop advanced damage to the GFB (43). This has led to a proactive search for more sensitive, specific, and reliable biomarkers to enable earlier prediction of DN onset and progression.

Novel biomarkers may be classified into glomerular, tubular, inflammatory, oxidative stress, and molecular categories. There is potential for investigating glomerular biomarkers, including transferrin and ceruloplasmin 44, and podocyte-specific biomarkers, such as nephrinuria, to precede microalbuminuria (41). Of interest are tubular biomarkers, which are damaged at an early stage in DN (44); these are NGAL, KIM-1, liver-type fatty acid-binding protein (L-FABP), and N-acetyl-2-D-glucosaminidase (NAG) (45). Inflammatory biomarkers, including their soluble receptors sTNF-αR1 and sTNF-αR2, adhesion molecules, and monocyte chemoattractant protein-1 (MCP-1), are significantly elevated in patients with DN, highlighting the critical role of inflammation in disease pathogenesis. Malondialdehyde (MDA), glutathione (GSH), superoxide dismutase (SOD), and catalase (CAT) are oxidative stress biomarkers that provide insight into the disequilibrium between pro-oxidant and antioxidant systems in DN (45).

**Figure 2:**
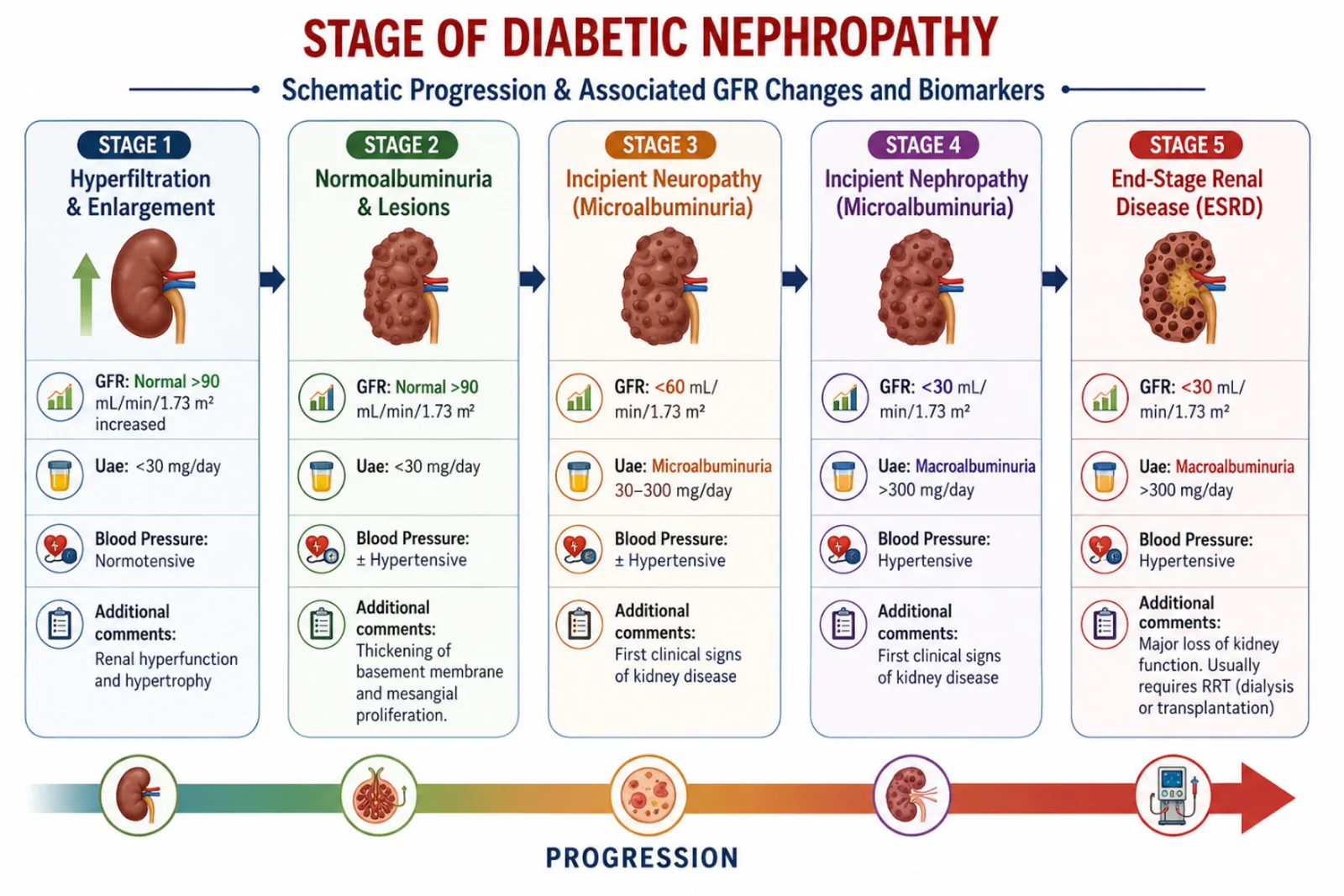
Schematic representation illustrating the sequential progression of diabetic nephropathy.

Other promising types of indicators include genetic, protein, and metabolic biomarkers, as well as noncoding RNAs (46). MicroRNAs, including miR-342-3p and miR-30a-5p, are elevated in patients with type 2 diabetes and micro- or macroalbuminuria, and exosomal miRNAs are being studied as urine biomarkers (46). Other potential candidates are cystatin C, which is correlated with eGFR and urinary podocyte destruction even in normoalbuminuric patients (47), and soluble urokinase plasminogen activator receptor (suPAR), which is associated with DN progression. KIM-1 and NGAL are also among the promising biomarkers of tubular injury and predictors of onset and progression (Figure 2). Several diagnostic biomarkers for DN are summarized in Table 1 (48).

**Table 1:** Key diagnostic biomarkers for DN.

S. No	Biomarker	Source	Clinical relevance	Stage of detection	Ref
1	Albuminuria	Urine	Classic DN marker; strongest predictor of kidney and CV risk, but not present in all DN cases	Early to advanced DN	(48)
2	Serum Creatinine/eGFR	Blood	Standard renal function marker; rises late when ∼40%–50% of kidney tissue is damaged	Mid to late DN	(48)
3	Cystatin C	Blood/Urine	More sensitive than creatinine, less affected by age/muscle mass; elevated even in normoalbuminuria	Early DN	(49)
4	NGAL	Urine/Plasma	Indicates tubular injury; elevated before albuminuria; sensitivity 94%, specificity 90%	Early DN	(50)
5	KIM-1	Urine	Sensitive marker of proximal tubular injury; linked to DN progression	Early DN	(51)
6	Vitamin D Binding Protein	Urine	Elevated in DN; linked to tubular damage and low vitamin D; sensitivity 98.8%	Early DN	(52)
7	Megalin (A- and C-megalin)	Urine	Receptor reabsorbing albumin; A-megalin ↑ in early DN, C-megalin ↑ in persistent microalbuminuria	Early DN	(53)
8	Transferrin	Urine	Appears before microalbuminuria; marker of early glomerular damage	Very early DN	(54)
9	Ceruloplasmin	Urine	Predicts microalbuminuria in normoalbuminuric T2D patients	Very early DN	(55)
10	Type IV Collagen	Urine	Structural GBM damage marker; increases with DN progression	Progressive DN	(56)
11	TNF-α-R1 & TNF-αR2	Blood	Strong predictors of renal function decline and ESRD; associated with early glomerular lesions	Early DN	(57)
12	MCP-1	Urine	Proinflammatory cytokine; elevated in macroalbuminuria; useful for prognosis	Progressive D N	(58)
13	TNF-α/ IL-6	Blood/Urine	Increased in micro- and macroalbuminuria; reflects inflammation	Progressive DN	(59)
14	8-oHdG (8-oxo-7,8-dihydro-2′-deoxyguanosine)	Urine	Oxidative stress marker; predicts DN progression and mortality	Progressive DN	(60)
15	NT-proBNP	Blood	Predicts decline of eGFR in early DN (BEAt-DN consortium)	Early DN	(61)
16	Periostin	Urine	High diagnostic precision; sensitivity 98%, specificity 80%	Early DN	(62)
17	Cyclophilin A	Urine	Early biomarker; sensitivity 84%, specificity 86%	Early DN	(63)
18	Uric Acid	Blood	Associated with insulin resistance, endothelial dysfunction, and microalbuminuria	Early DN	(64)
19	Proteomic Classifier (CKD273)	Urine	Proteomic panel predicting DN progression (PRIORITY trial)	Early DN	(65)
20	MicroRNAs (miR-192-5p, miR-130b)	Urine/Plasma	Potential early markers for DN detection and monitoring	Early DN	(66)
21	Lipidomics markers	Blood	Alterations in lipid pathways (e.g., phosphatidylethanolamine, TAGs) associated with DN severity	Early to progressive DN	(67)
22	Genomic Biomarkers (ELMO1, FRMD3, IL-6/IL-10 polymorphisms	DNA	Indicate genetic susceptibility and inflammatory pathways	Early DN	(67)
23	Transcriptomic Biomarkers (miR-21, circRNA-ATG7)	Urine/Plasma	Regulate fibrosis, autophagy, and inflammation; potential for early DN detection	Early DN	(68)
24	Metabolomic Biomarkers (TMA, TMAO, amino acids, lactate)	Urine/Plasma	Reflect hypoxic stress and metabolic dysregulation in DN	Early to progressive DN	(69)
25	L-FABP (Liver-type Fatty Acid Binding Protein)	Urine	Reflects proximal tubular injury and oxidative stress; rises before albuminuria	Early DN	(70)
26	α-1 Microglobulin	Urine	Early biomarker of tubular injury; predicts microalbuminuria in T2D	Early DN	(71)
27	Pentosidine (AGEs)	Blood/Urine	Marker of glycation and collagen cross-linking; linked with DN fibrosis	Progressive DN	(72)

Although such progress has been made, the majority of new biomarkers still need to be validated in larger clinical trials before they can be used routinely (44). Finally, it aims to develop a panel of biomarkers that meets the sensitivity and specificity criteria, enabling higher detection rates, fine-grained risk assessment, and timely intervention to prevent DN from progressing to ESRD (44, 46).

## 3 Diagnostic approaches

**Figure 3:**
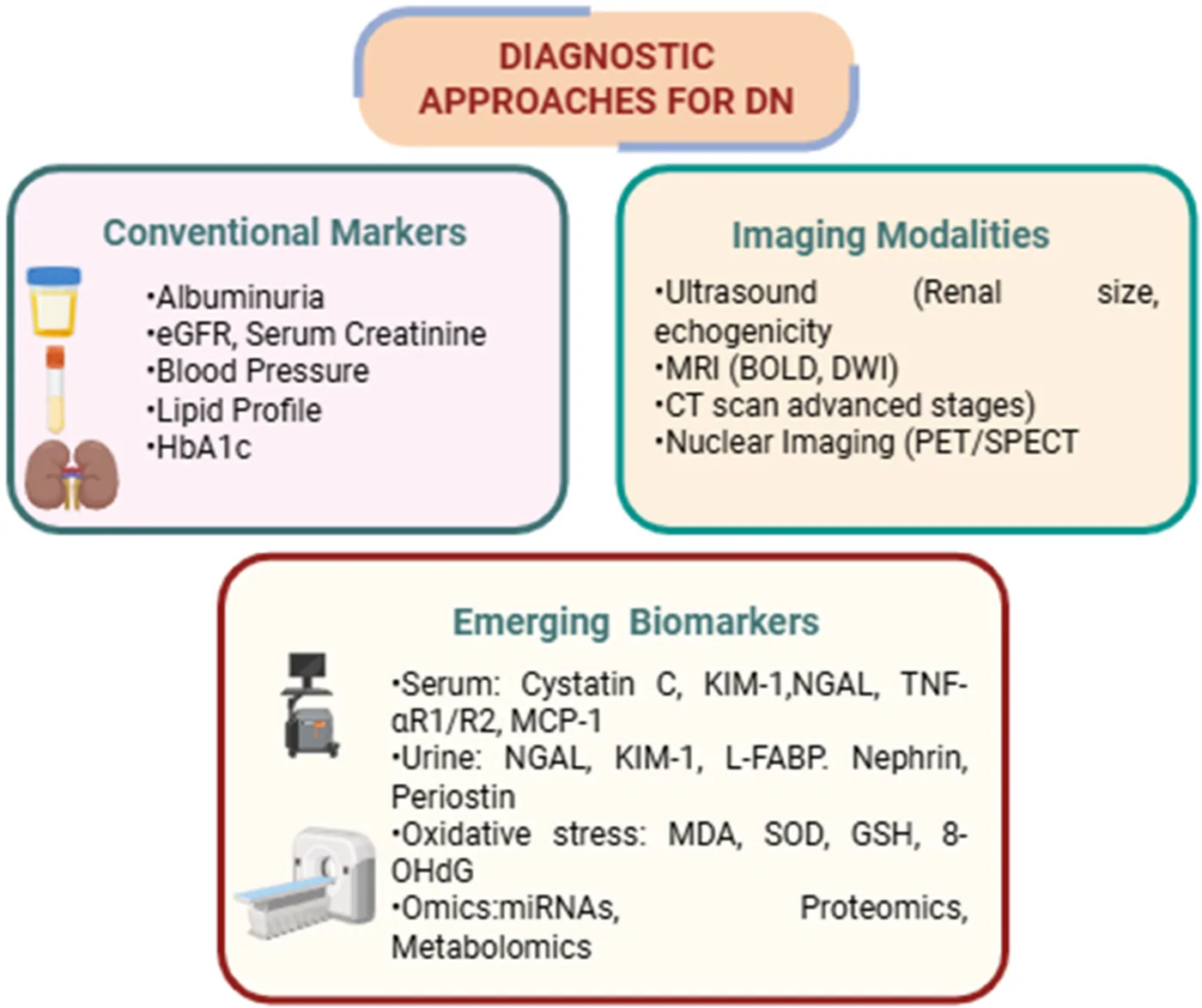
Current diagnostics approaches for DN.

To improve the lives of people with DN and to decrease the impact on society, primary identification of progression of DN with suitable screening and diagnostic tools is very significant in order to deliver timely and appropriate management (Figure 3) (73).

### 3.1 Conventional diagnostic tools

Identification and monitoring of DN rely mainly on estimated eGFR and albuminuria, which serve as standard clinical markers for kidney function and damage, and there are several stages of DN. Respectively, while widely used to guide early interventions and assess risks of cardiovascular disease and end-stage renal disease, these markers have notable limitations for identifying and monitoring DN, highlighting the need for improved biomarkers for effective disease management (73).

#### 3.1.1 Albuminuria

Albuminuria is regarded as a sensitive biomarker for CKD and cardiovascular disease risk and serves as the initial clinical sign of DN (74). The measurement of albumin levels in a 24-hour urine collection is regarded as the definitive standard for diagnosing DN. But in everyday life, it's hard to get a 24-hour urine sample. Also, this method doesn't give exact or correct facts. The guidelines suggest using the albumin to creatinine ratio of a spot urine sample as a stand-in for the volume of urinary albumin in a 24-hour urine collection. This is a simple approach that may be carried out in a clinical environment (75, 76). In order to overcome the fluctuation in urine concentrations induced by hydration, concurrent measurement of spot urine albumin and creatinine values is commonly used as a marker for the screening of albuminuria. This allows these values to be normalized. As per the albumin-to-creatinine ratio, patients are divided into 3 categories of albuminuria by Kidney Disease Improving Global Outcomes: A1 is Normal-to-mildly increased albuminuria (<30 mg/g), A2 moderately improved albuminuria (30–300 mg/g), and A3 is severely increased albuminuria (>300 mg/g) (77, 78). According to the American Diabetes Association, a patient is said to have albuminuria if at least 2 out of 3 urine ACR values that are checked within 6 months are abnormal (76). A decrease in GFR is linked to the development from category A2–A3, which is expected to occur at a rate of 2%–3%. Urinary ACR (A2) that is consistently and moderately elevated is a known predictor of elevated cardiovascular disease risk and a sign of the onset of diabetic renal disease (79, 80).

#### 3.1.2 eGFR

Although GFR is merely one aspect of renal excretory function, it is widely regarded as the greatest measure of kidney function overall since it typically declines following extensive structural damage, and most other kidney functions in CKD drop along with GFR (78). Equations like Modification of Diet in Renal Disease (MDRD) study are used widely for the estimation of GFR, and serum creatinine is used to estimate these GFRs (81, 82). Conventionally, based on eGFR the severity of CKD has been divided into 5 phases G1, GFR ≥90 ml/min/1.73 m^2^; G2, 60–89 ml/min/1.73 m^2^; G3, 30–59 ml/min/1.73 m^2^, G4, 15–29 ml/min/1.73 m^2^ and G5 < 15 ml/min/1.73 m^2^ (83, 84). To address these limitations, alternative markers have been explored, with cystatin C- a cysteine protease inhibitor freely filtered by the glomeruli and metabolized in the proximal tubule- emerging as an auspicious indicator of renal impairment (85).

### 3.2 Emerging biomarkers

Due to the limitations of creatinine and albuminuria, along with the occurrence of normoalbuminuric phenotype in DN, additional diagnostic tests are required to accurately detect the disease.

#### 3.2.1 Serum cystatin C

Cystatin C is a type of low molecular protein of 122 amino acids belonging to the cysteine protease inhibitor family. The CST3 housekeeping gene encodes it and is situated on chromosome 20 (86). Several physiological functions are involved by cysteine cathepsins, such as protein degradation, protein processing, antigen presentation, bone remodeling, and apoptosis (87). Cystatin C is the most prevalent and powerful endogenous inhibitor that regulates these enzymes within and outside of cells, involved in various pathological conditions like inflammation and cardiovascular disease (88). Cystatin C is produced by nucleated cells in the body and secreted into the plasma at a steady rate. Owing to its small molecular size and positive charge, it is freely filtered through glomeruli and subsequently reabsorbed and destroyed without being secreted in the renal tubule (89, 90). Consequently, serum cystatin C serves as a biomarker for early detection of AKI and can indicate initial alterations in renal function and declines in eGFR. Its sensitivity and specificity are expected to be 71% and 53% within the first 6 h after the cardiovascular surgery (91). Although cystatin C allows earlier identification of AKI compared to creatinine changes, its diagnostic utility is still limited when compared against other indicators like NGAL (92, 93).

#### 3.2.2 Neutrophil gelatinase-associated lipocalin (NGAL)

NGAL is a 25 kDa type of protein that belongs to the lipocalin family. It is synthesized by neutrophils and damaged nephron epithelial cells. In reaction to nephron injury, it is specifically secreted into blood and urine (94). NGAL is filtered from plasma through the glomerular filtrate and then reabsorbed by endocytosis through the megalin system in the proximal tubule. A study suggests that NGAL levels were 1.5 times greater when the levels of serum and urinary damage markers were measured in diabetic patients compared with the non-diabetic patients. Independent of eGFR, NGAL and other indicators of tubular and glomerular damage correlate with albuminuria, suggesting that both glomerular and tubular injury play a role in its development (95). NGAL can identify early DN changes before proteinuria, and there is evidence that tubular injury may occur before glomerular damage. Its function as an early biomarker for DN is supported by the fact that both serum and urine NGAL have been demonstrated to predict the development of albuminuria (96).

#### 3.2.3 Plasma KIM-1

Kidney Injury molecule KIM-1, initially discovered as hepatitis A virus receptor (HAVCR1, often referred to as TIM-1) (97), is a type 1 transmembrane glycoprotein found in the apical membrane of proximal renal tubular cells. Its levels in the blood tend to rise in people with tubular injury (98). In a cohort study of type 1 diabetes patients with proteinuria, baseline serum KIM-1 levels were revealed to be a robust predictor of eGFR decline and progression to ESKD over a five - to fifteen-year follow-up period even after adjustment for baseline urinary albumin-to-creatinine ratio, eGFR, and HbA1c (99). Moreover, KIM-1 levels were demonstrated to predict early eGFR reduction and kidney disease progression in a sample of 462 patients, including 259 with normal albuminuria and 203 with microalbuminuria, regardless of systolic blood pressure, HbA1c, AER, eGFR, and TNF-αR1 (100). Conversely, evidence regarding urinary KIM-1 in DN has so far been limited and less promising (98).

### 3.3 Role of imaging in nephropathy diagnosis

Imaging plays an important role in the diagnosis, evaluation, and monitoring of nephropathy, especially in patients with diabetes or other CKD. Although biochemical markers like albuminuria, eGFR, cystatin C, NGAL, and KIM-1 remain important for early detection, imaging helps in structural and functional assessment. Advanced imaging, including Magnetic Resonance Imaging (MRI), Ultrasound, Computed Tomography (CT), and scintigraphy (PET, SPECT).

#### 3.3.1 MRI

MRI is a non-invasive technique for describing structural and functional changes in nephropathy beyond the constraints of traditional biomarkers. Specifically, two sophisticated MRI methods, including blood oxygen level-dependent (BOLD) and diffusion- weighted imaging (DWI), are safe for evaluating renal microstructure and function (101). BOLD MRI measures intrarenal oxygenation levels using deoxyhemoglobin as an endogenous contrast agent, whereas DW MRI measures the diffusion of water molecules in the renal tissue to calculate the average apparent diffusion coefficient. Remarkably, substantial inverse relationships have been reported between kidney ADC values and the extent of renal fibrosis in CKD. Furthermore, other studies have suggested that MRI methods may provide better diagnostic precision than albuminuria for detecting DN (102, 103).

#### 3.3.2 Two-dimensional ultrasound radiomics model

USG is commonly used for assessing kidney size, parenchymal thickness, and echogenicity in clinical practice. These morphological measures have low sensitivity for early disease detection, although they are helpful in advanced stages of DN. To fill this gap, recent research has applied machine learning and radiomics techniques in ultrasound imaging, which allow for the quantitative extraction of picture aspects that go beyond visual interpretation. In 2023, SU et al. developed a 2D ultrasound radiomics model for the early DN evaluation. The researchers created a machine learning diagnostic system combining the high dimensional characteristics of grayscale renal ultrasonography images and biochemical markers. The model demonstrated excellent diagnostic accuracy in both training and test batches with a K-nearest neighbor (KNN) classifier, and an AUC of 0.94 and 0.91, respectively. These findings illustrate the radiomics characteristics that capture textural and intensity- based patterns that are not perceptible to the human eye and have a strong discriminatory power for early detection of DN. Hence, when combined with biochemical measures such as serum creatinine and albuminuria, this approach is a more powerful tool for risk stratification to give a more accurate picture of renal involvement in diabetes (25).

### 3.4 Limitations of current diagnostic strategies

The available diagnostic facilities, such as clinical biomarkers, imaging, or histopathology, remain challenging for DN. Even while methods such as urinary albumin excretion, eGFR, and renal biopsy serve as the foundation for diagnosis and staging, each has substantial limitations that prevent early and precise disease detection. Despite being a commonly used early sign, albuminuria lacks specificity since it can be found in other renal disorders and cannot detect non-proteinuric DN. Moreover, the levels might fluctuate owing to temporary reasons, including exercise or infections (104). The accuracy of eGFR decline is affected by age, sex, muscle mass, and ethnicity, and it usually manifests later after significant nephron loss (105). The renal biopsy is still the gold standard for conclusive diagnosis and differentiation from non-diabetic kidney disease, while being intrusive, risky, and inappropriate for routine monitoring (106). The use of conventional modalities, including MRI, CT, and ultrasound, is primarily beneficial in advanced disease diagnosis. CT is limited by radiation and contrast-induced nephropathy, ultrasound is limited by only gross morphological changes, MRI shows promise with functional sequences like BOLD and DWI, but is limited by cost, accessibility, and lack of standardized thresholds (102). Together, all the methods hinder the detection of novel biomarkers such as KIM-1, NGAK, and cystatin C, which have the potential to be used in early diagnosis of tubular damage, but they are not extensively used or standardized in clinical practice (107). The DN at the early stage due to a lack of sensitivity, invasiveness, and limited clinical applications.

## 4 Current management strategies

**Figure 4:**
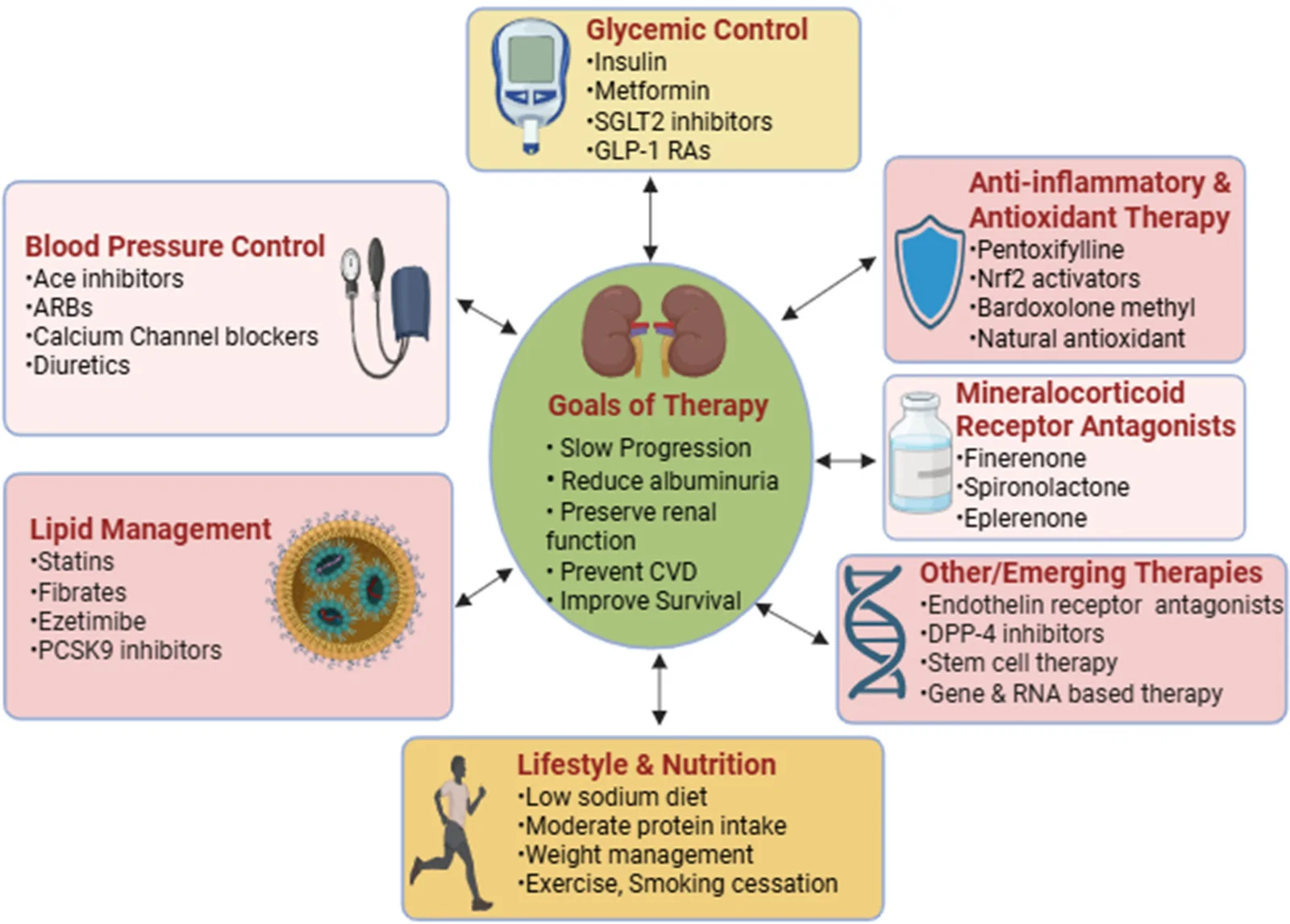
Current therapeutic approaches in DN.

Current management strategies for the DN primarily focus on glycemic control, blood pressure control, lipid control, anti-inflammatory and antioxidant therapies, lifestyle and nutritional interventions, as shown in Figure 4, Table 2 (108, 109).

**Table 2:** Overview of the existing pharmacological treatment approaches employed in the management of DN.

S. No	Drug class	Example	Mechanism of action	Clinical outcome	Ref
1	SGLT2 inhibitors	Empagliflozin, Dapagliflozin, Canagliflozin	Inhibit renal SGLT2 → reduce proximal tubular glucose reabsorption → glycosuria, lower intraglomerular pressure (↓hyperfiltration)	Reduce albuminuria, slow eGFR decline and lower risk of kidney-failure outcomes in CKD with/without diabetes (benefit shown across CREDENCE, DAPA-CKD, EMPA-KIDNEY program).	(110)
2	GLP-1 receptor agonists	Semaglutide, Liraglutide	GLP-1 receptor agonism → improved glycemic control, weight loss, anti-inflammatory and hemodynamic effects	Reduced progression of clinically important kidney outcomes and CV death in T2D with CKD; kidney benefits reported in large outcome trials (e.g., FLOW/semaglutide analyses).	(111)
3	ACE inhibitors/ARBs (RAAS blockers)	Enalapril, Lisinopril, Losartan	Inhibit angiotensin II formation or block AT1 receptor → efferent arteriolar vasodilation, reduced intraglomerular pressure, anti-fibrotic effects	Consistently reduce proteinuria and delay progression of DN; cornerstone therapy for DN	(112)
4	Non-steroidal MRA	Finerenone	Mineralocorticoid receptor antagonism with greater selectivity → anti-inflammatory and anti-fibrotic renal effects	Reduced risk of CKD progression and cardiovascular events vs. placebo in patients with T2D and CKD 5(FIDELIO-DN/FIGARO-DN results).	(113)
5	Endothelin receptor antagonists	Atrasentan	ETA receptor blockade → reduces proteinuria via modulation of glomerular hemodynamics and anti-fibrotic actions	Significant albuminuria reduction; clinical-event benefit limited by fluid retention/heart-failure risk (SONAR and follow-up analyses).	(114)
6	Nrf2 activator/antioxidant	Bardoxolone methyl	Activates Nrf2 → antioxidant, anti-inflammatory gene expression; may improve GFR via hemodynamic/mitochondrial effects	Early trials showed eGFR rises but major trial (BEACON) stopped for increased CV events; efficacy/safety profile remains uncertain and under further study.	(115)
7	Anti-inflammatory/phosphodiesterase inhibitor	Pentoxifylline	Nonselective phosphodiesterase inhibitor → anti-TNF-α and anti-inflammatory effects that reduce glomerular inflammation	Multiple small trials/meta-analyses suggest reductions in proteinuria; larger high-quality trials needed to confirm impact on long-term CKD progression.	(116)
8	DPP-4 inhibitors	Linagliptin	Inhibits DPP-4 → increases endogenous incretins (GLP-1, GIP) → glucose lowering with neutral renal hemodynamic effects	Generally safe in CKD; trials (e.g., CARMELINA) showed neutrality for major kidney outcomes but some reduction in albuminuria progression.	(117)
9	Lifestyle/Dietary interventions	–	Dietary sodium restriction, protein moderation, weight loss, exercise → improve BP, reduce albuminuria and metabolic stress	Non-drug measures that complement pharmacotherapy; can reduce albuminuria and slow progression when combined with optimal medical therapy.	(118)
10	Mesenchymal Stromal/Stem Cells (MSCs)	Human MSCs	Immunomodulation, anti-inflammatory, anti-fibrotic; restoration of autophagy; paracrine effects (exosomes)	In a murine model (DN) a single IV injection of human MSCs (at both early and later stages of disease) led to improved kidney function, reduced albuminuria, decreased fibrosis & inflammation, and restoration of autophagy pathways.	(119)

### 4.1 Glycemic control

Poor glycemic control acts as an independent risk factor for the development of albuminuria and progression of ESRD in DM (105). Various clinical studies have shown that elevated glucose concentrations are an initial setting for diabetic complications, including renal disease. In chronic hyperglycemia, a transient spike in blood glucose may have a long-lasting impact on DN development (120, 121). Thus, standard treatment for diabetic nephropathic patients still focuses on glycemic control, and some major clinical trials have shown the benefits of strict glycemic control in preventing DN during early diabetic mellitus. So current approaches prioritize the prudent use of Insulin, Metformin, SGLT2, and GLP-1 receptor agonists. Insulin is the most effective treatment to reduce glucose in type 1 and type 2 diabetes, along with CKD patients, while the use of oral drugs is limited. While using the insulin, it offers dependable glycemic control and flexible dose adjustments, but hypoglycemia risk rises as kidney function declines because of decreased insulin clearance, requiring cautious titration and regular monitoring (122). Oral agents like metformin are advised for type 2 diabetes treatment, and they offer weight neutrality and effective cardiovascular benefits. However, its use must be tailored because of lactic acidosis, along with dose reduction in moderate CKD, and discontinue once eGFR falls below 30 ml/min/1.72 m^2^ (123). The use of SGLT2 inhibitors has transformed the DN treatment via decreasing the intraglomerular pressure, proteinuria, and progression of CKD, and the clinical trials have shown strong reno-protective CVS benefits that are independent of glycemic control. Other antidiabetic drugs, such as GLP-1 receptor agonists like liraglutide and semaglutide, improve glycemic control along with weight loss and decrease albuminuria (124). Together, these treatments form a complementary regimen that reflects the paradigm shift in the management of DN toward approaches that go beyond glycemic control. The insulin offers flexible glycemic control, metformin aids in early-stage management, SGLT2 inhibitors and GLP-1 receptor agonists provide both metabolic and organ protective effects.

### 4.2 Blood pressure control

#### 4.2.1 Use of ACE inhibitors and ARBs

DN is associated with hypertension. Controlling blood pressure in rats with DM has been shown in one animal model to delay the onset of glomerulosclerosis and albuminuria. RAAS is the most studied mechanism of blood pressure regulation, and the treatment that targets the RAAS plays a key role in the management of DN (103). RAAS inhibitors, including ACEIs and ARBs, have been used for several years for the treatment of DN, and studies have shown that RAAS inhibitors are useful in renal function protection (125). It has been shown that inhibition of ACE reduces systemic blood pressure, albuminuria, and glomerular capillary pressure (126). ACE inhibitors show their effects via inhibition of ACE, along with interruption of vasodilating substances like bradykinin, and restore glomerular capillary pressure and reduce microalbuminuria (127). By using ACE inhibitors, the progression of DN can be slowed down in nephropathic patients with early examinations and treatment. ARBs decrease blood pressure by reducing vasoconstriction, aldosterone excretion, and promoting vasodilation of efferent arterioles of the glomerulus. These drugs prevent angiotensin II by directly blocking the angiotensin II receptor, thereby decreasing the negative effects of angiotensin II on renal hemodynamics. ARBs help to reduce the development of DN through decreasing the systemic blood pressure and reducing UAE. Numerous trials have shown ARBs to be effective in slowing the development of DN (126, 128).

#### 4.2.2 Role of calcium channel blockers and diuretics

Calcium channel blockers are divided into 2 subtypes: the dihydropyridines (like amlodipine, felodipine) and non-dihydropyridines (Verapamil and Diltiazem). The non-dihydropyridine includes Verapamil and Diltiazem, which are more cardioselective and are beneficial and as effective as ACE inhibitors in terms of their actions and effects on DN and proteinuria. This sub-group has been shown to decrease proteinuria significantly when used in combination with an ACE inhibitor and improve renal outcomes in patients with DN (129, 130). However, the effects of dihydropyridines like amlodipine and felodipine on proteinuria and DN have been inconsistent. DN. While some can enhance albuminuria, with some having no effect, and some causing improved albuminuria. This variation is related to their different effects on intrarenal activity and their sites of action within glomeruli (131).

Numerous studies have shown that thiazide diuretics, including hydrochlorothiazide, reduce albuminuria in DN patients, which may be due to lowering intraglomerular pressure. Though other studies suggest that the renoprotective effects might be different from their antihypertensive effects (132, 133). In a study, the effects of hydrochlorothiazide and sodium restriction on albuminuria were evaluated in 45 patients with DM. The results demonstrated that hydrochlorothiazide and sodium restriction significantly decreased albuminuria (134).

### 4.3 Lipid-modulating agents

Lipid-lowering agents improve lipid growth, reduce renal fibrosis, and inflammatory mediator expressions (135). In early DN, the application of fibrates and statins has positive effects to reduce albuminuria and slow the fall of eGFR. Statins are HMG-CoA reductase inhibitors, which decrease the levels of serum LDL, HDL, and triglycerides. A study by Li et al. found that in DN patients, atorvastatin reduces renal function damage, lowers serum inflammatory factor levels, and improves hemorheology (136). The 2 meta-analyses' findings suggested that in DN patients, statins have positive effects on renal function in a time-dependent manner with a substantial reduction in albuminuria between 1 and 3 years of statin treatment (137, 138). Furthermore, one more meta-analysis of 3,426 individuals with early DN found that statins might raise eGFR, decrease serum creatinine and C-reactive protein levels, which reduces the inflammatory response and protects the kidney (139).

Fibrates are agonists of peroxidase proliferator-activated receptor, which decrease the TG levels and increase HDL levels. A fibrate agonist's fenofibrate treatment was linked with a decreased incidence of microalbuminuria and macroalbuminuria along with a lower rate of eGFR decline in DM, as per *post hoc* analysis of the ACCORD study (140).

### 4.4 Anti-inflammatory and antioxidant therapies

Several reports have suggested that inflammatory cells like leukocytes, monocytes, and macrophages are linked to the development of DN, and inflammatory factors, including ILs and TNF-α, in both the kidneys of animals and humans with DM are upregulated (141, 142). Furthermore, an animal study has confirmed a protective effect of preventing the entry of inflammatory cells into the kidneys (143). In DN patients, the accumulation of advanced glycosylation end products can activate can NF- κB that raises the transcription levels of proinflammatory factors and chemokines such as TNF- α, IL-1β, and IL-6, exacerbating the kidney inflammation injury (144). A methylxanthine derivative, pentoxyfylline, acts as a non-specific phosphodiesterase inhibitor, activating protein kinase A and suppressing the production of inflammatory functions like IL-6 and TNF- α (145).

Oxidative stress caused by the development of ROS because of cellular respiratory failure in diabetic circumstances plays a critical role in the DN progression (146). Numerous studies, including animal and human DN patients, have demonstrated the beneficial effects of antioxidants on DN patients by various molecular mechanisms. Nrf2 (nuclear factor erythroid-derived 2)-like 2 is an important regulator that protects the cells and antioxidants, and is mainly activated in response to oxidative stress (147). In a study, Streptozotocin (STZ) induced rat DN models in which serum MDA levels were a classic DN marker; the strongest predictor of kidney and CV risk, but not present in all DN cases, preventing systemic complications of diabetes. The diabetic patients are required to control blood glucose, lipids, blood sugar, and hypertension (118). For DN patients, dietary adjustments are advised for carbs, protein, fats, and electrolytes, with the latter being based on each person's renal function (148, 149). For the prevention and management of diabetes and DN along with their complications, including CVD, dyslipidemia, and hypertension, weight control and physical exercise are key modifiable risk factors. Nutritional therapy, which necessitates a precise assessment of vitamin and electrolyte requirements, is supplemented by prescribed activity. Additionally, new research emphasizes the additional therapeutic benefits of particular food patterns, strengthening nutrition therapy for DN. The determination of nutrition intervention, such as macronutrient, micronutrient, and electrolyte are required for DN. The evidence suggests that some dietary patterns may provide additional therapeutic benefits (150, 151).

### 4.5 Lifestyle and nutritional interventions

The multifactorial pathogenesis of DN makes the point of a management approach that relies on dietary and lifestyle modifications as well as on a pharmacological treatment. These interventions aim to improve glycemic and blood pressure control, minimize metabolic burden on the kidneys, and improve cardiovascular health.

#### 4.5.1 Lifestyle modification

The lifestyle interventions are crucial in DN management as they address multiple risk factors at once. Exercise, especially aerobic and resistance training, enhances insulin sensitivity, decreases systemic inflammation and oxidative stress all of which help to protect the renal. In overweight and obese individuals after significant weight loss, there were notable improvements in glycemic control, blood pressure and albuminuria levels, all of which are associated with an increase in renal injury progression (152). Smoking cessation is important because it can slow the progression of glomerular sclerosis, decrease the bioavailability of nitric oxide and affect oxidative stress. Similarly, keeping a sound sleep and using stress reduction techniques such as mindfulness or yoga has been associated with improved metabolic stability and decrease sympathetic activation that indirectly improves renal health (153). In recent years, digital health interventions have proven useful additions to traditional lifestyle management. Mobile-based self-management programs allow continuous monitoring, education and feedback, resulting in better adherence to lifestyle goals. A particular study was DialBetesPlus, where diabetics with type 2 showed a significant reduction in urine albumin excretion when monitoring their diet, activity and blood glucose levels via an mHealth application. These technology-based methods could address some of these care gaps and provide personalized and real-time assistance for patients with DN (154).

#### 4.5.2 Nutritional interventions

Proper nutrition is an integral component of the treatment of DN: nutrition directly affects glycemic control and hemodynamic stability and an increase in metabolic stress in the kidneys. New research confirms that the shift to a balanced diet rather than single nutrient restriction may be more sustainable and effective in slowing the DN development (155). Both the most research-backed approaches – such as the Mediterranean and Dietary Approaches to Stop Hypertension diets – focus on eating more vegetables, fruits, whole grains, legumes, nuts, olive oil and lean protein sources while limiting the amount of refined carbohydrates, red meat and processed foods. The diets are rich in antioxidants, unsaturated fatty acids, and anti-inflammatory phytochemicals, which decrease oxidative stress and endothelial dysfunction in DN pathogenesis (152, 156). The nutritional care for DN aims at decreasing renal stress and improving the metabolic process. The recommended dose of sodium is <2 g/day to help control the blood pressure and albuminuria with an RAAS inhibitor. Consumption of protein should be individualized with moderate restriction (0.8 g/kg/day) that helps to decelerate the fall of GFR without the risk of malnutrition. But when it comes to the quality of carbohydrates like grains, dietary fiber, and resistant starch, they increase insulin sensitivity, regulate gut microbiota, and protect the kidneys. When adapted under dietary guidance, these measures play a role in maintaining kidney function and combine with pharmacologic therapy (152, 153).

## 5 Challenges in conventional therapies

Difficulties with Traditional treatments Although much progress is being made in the management of DN, conventional pharmacological interventions still have their drawbacks (157). There are current strategies that can slow disease progression, including strict glycaemic control, RAAS blockade, lipid-lowering agents and newer classes of medications such as SGLT2 inhibitors and GLP-1 analogues; however, none of these therapies are able to stop or reverse kidney damage (157). There are still a number of intrinsic limitations which limit their effectiveness and potential for use in the long term.

### 5.1 Poor bioavailability and systemic toxicity

The bioavailability is poor and systemically toxic. The aqueous solubility of many therapeutic agents is poor, they are easily degraded, and they are extensively metabolized in the first pass. For instance, natural antioxidants like curcumin and resveratrol, which are effective in preclinical DN models, have low bioavailability and systemic clearance very quickly, leaving very low renal exposure (158, 159). Furthermore, these systemic doses are frequently high to reach adequate bioavailability and can result in hepatotoxicity, off-target organ damage and gastrointestinal side effects. Likewise, ACE inhibitors or ARBs may cause hyperkalaemia, hypotension and acute kidney injury in the long term, limiting their tolerance in high-risk patient groups (158).

### 5.2 Lack of targeted drug delivery

Unspecific distribution of drugs. There is little specificity with drug regimens that are conventional, and they are distributed throughout the body, but not necessarily to the kidneys. This results in sub-optimal concentration of drug in kidney and unnecessary high concentration in other organs. For instance, anti-inflammatory, anti-fibrotic drugs exert a systemic effect on the tissues, reducing their therapeutic effects in the glomeruli or tubulointerstitial spaces (160). Moreover, the GFB and efflux transporters impede drug penetration in the kidney, further complicating targeted delivery. This is one of the reasons why these are not very effective and can have side effects on the whole organism (161).

### 5.3 Patient compliance issues

Patient compliance issues Long-term polypharmacy is frequently necessary in the successful management of DN, such as lipid-lowering agents, antihypertensives, renoprotective therapy, and antidiabetic agents. Many patients are not adherent to this multidrug regimen, in part due to its complexity and due to dietary and lifestyle changes required (162). Other side effects associated with the use of metformin (digestive problems), SGLT2 (recurrent genitourinary infections), and insulin (frequent hypoglycaemic events) further decrease patient adherence. Non-adherence can negatively impact therapeutic effectiveness and can lead to the rapid progression of the disease to ESRD (163–165).

### 5.4 Renal-specific drug clearance limitations

Limiting factors for renal clearance of drugs. The kidney is a key excretory organ, presenting special problems in pharmacotherapy. Drugs or metabolites can be rapidly cleared from the kidney, which can reduce the time spent in the nephron, requiring a higher or more frequent dose (166). On the other hand, if the kidneys are not working well, certain drugs can build up to dangerous levels, necessitating frequent dosage adjustments and monitoring (167). For example, in the context of advanced CKD, lactic acidosis can occur as a side effect of metformin, and there are some lipid-lowering and anti-inflammatory drugs that have altered pharmacokinetics in reduced GFR states. To complicate matters further, these variations can pose difficulties for personalised dosing strategies and reduce the safety margin of traditional treatments (168).

## 6 Nanotechnology in DN

The Nanotechnology of DN offers new hope for the cure of diabetes and its associated complications through precise, site-specific drug delivery. Nanotechnology is a promising discipline because of the development of nanostructures that can be applied both therapeutically and diagnostically in the treatment of diabetes (18). According to recent findings, nanosystems are a potential theranostic platform likely to be used in future DN diagnosis and treatment (158, 159). Due to their ease and safety, nanotechnology-based technologies have been proposed as a more acceptable alternative to the administration of insulin and other hypoglycemic drugs.

### 6.1 Overview of nanocarriers

Nanocarriers are small, nanoscale particles with unique physical, chemical, and biological properties that make them useful tools in diagnostics and treatment (159). The benefits they have include improved pharmacokinetic characteristics, stability, bioavailability, trackability, biocompatibility, and the therapeutic efficacy of strong chemotherapeutic drugs, just to mention a few (159).

#### 6.1.1 Liposomes

The liposomes have great potential as drug carriers because they are biodegradable, biocompatible, exhibit increased retention in the kidneys, and have low immunogenicity. They have benefits such as enhanced membrane permeability, sustained release, and site-specific delivery through surface functionalization (159). PEGylation, however, could provide a solution for stability and circulatory longevity *in vivo* and help liposome systems address their greatest long-term stability challenges. For example, at the early stages of DN, liposomes containing coenzyme Q10 and ultrasound-induced microbubble destruction enhance renal hemodynamics. This method resulted in the preservation of podocytes and prevention of apoptosis, and low to no cytotoxicity (159). The second study showed that better delivery of co-enzyme Q10 to the kidneys was achieved by encapsulating it in liposomes and by destroying ultrasound-targeted microbubbles, as a means of site-specific delivery (169). Since nanoparticles less than 10 nm are rapidly excreted by the kidneys, optimal nanoparticle sizes are an especially critical factor. Nevertheless, the immune system identifies and removes NPs with diameters greater than 150 nm (158). A study on Glutathione-loaded liposomes (GSH-LIP) showed potential as a new antioxidant and AR-inhibitor treatment, as it enhanced glutathione bioavailability, ROS scavenging, aldose reductase activity, and accumulation in the sorbitol polyol pathway, and was selective for the kidneys to control renal pathology in DN (170). By binding to GULT transporters, mannose-modified liposomes were formulated to target diabetic kidneys, thereby therapeutic effectiveness. These alterations highlight the ease of customizing liposomes as nanomedicine vectors to treat diabetes (171). A new hirudin/liposome complex has also been found to have therapeutic effects in nephropathy among diabetic patients. Liposomal encapsulation of hirudin in a DN rat model showed a significant increase in renal accumulation of hirudin compared with free drug administration, leading to less renal damage. In terms of mechanisms, the combination inhibited the production of TGF-1 and VEGF in renal tissue, which are important mediators of angiogenesis and fibrosis (171). These results show that liposomal delivery should be considered an efficient way to regulate pathogenic signaling pathways, supporting the use of liposomes as a nanocarrier platform in DN therapy. All these studies point to the utility of liposomes in DN therapy. Liposomes are an effective nanoplatform for improving drug stability, enhancing kidney targeting, and regulating pathogenic pathways crucial to DN through antioxidant and antifibrotic interventions (172).

#### 6.1.2 Solid lipid nanoparticles (SLNs)

SLNs, once termed lipospheres, are a potential pharmacological nanocarrier for controlled drug release. From 50 nm to 500 nm, they are smaller. SLNs consist of physiologically compatible solid lipids that are stable at room and body temperature and are stabilized by surfactants. They have a lipid matrix that could be used to incorporate lipophilic and, depending on the formulation design, hydrophilic therapeutic agents, resulting in controlled drug delivery, increased resistance to degradation compared to conventional dosage forms (173). Suboptimal bioavailability, the need to regulate drug delivery, and reduced dosing requirements due to suboptimal drug bioavailability are some of the shortcomings of conventional drug delivery systems that are effectively countered by SLNs, which incorporate several beneficial properties of polymeric nanoparticles (173). Micritrin-loaded SLNs have been shown to enhance DN changes more conspicuously by reducing oxidative stress and increasing antioxidant enzyme levels compared with native myricitrin, thereby eliminating bioavailability problems (174).

#### 6.1.3 Metallic and inorganic nanoparticles

Although, in principle, metallic and inorganic nanoparticles can be advantageous for therapeutic and targeting applications, they should not be regarded as inherently safe or advantageous nanocarriers in general, and especially for patients with chronic kidney disease or diabetic nephropathy. They have biological effects that are highly dose-, duration-, size-, and composition-dependent. Dissolution/release of these metal ions, particularly from nanoparticles such as AgNPs and ZnO NPs, can contribute to the generation of ROS, oxidative stress, mitochondrial dysfunction, inflammation, and genotoxicity. Furthermore, if the nanoparticles are not efficiently cleared from the tissues, they can bioaccumulate and have chronic exposure in tissues that can cause long-term renal and systemic toxicity (175). The therapeutic window may then be relatively small, and therapeutic concentrations may approach those associated with organ-specific toxicity. Therefore, the safety and the potential for translation of this class of metallic nanocarriers should be assessed on a formulation-specific basis, including metal ion release, biodistribution, biodegradation/clearance, dose-response, and long-term toxicity, and not taken for granted.

#### 6.1.4 Polymeric nanoparticles (PNPs)

PNPs are used with a high rate of drug loading due to high drug-loading capacity, excellent biodegradability, and biocompatibility. Examples of common nanocarriers and PNPs include polymeric micelles, liposomes, dendrimers, polymeric sponges, and colloidal carriers. Also, these nanoparticles exhibit lower toxicity, higher therapeutic activity, controlled drug release, greater physical and chemical stability, and improved drug penetration. PNPs often have been used as drug delivery vehicles because of their ability to transport synthetic and natural molecules, including medicines, growth factors, proteins, peptides, DNA, and mRNA (176). Polymeric nanoparticles are of significant interest to scientists because they are highly efficient, can be manufactured easily, have a long circulation time, are biocompatible and non-toxic, have the capacity to absorb or encapsulate other molecules, and can also be actively targeted through surface modification (177). Polymeric nanoparticle applications, particularly PLGA-based systems, have demonstrated potential as a means to treat diabetic neuropathic pain. By improving glycemic control with oral insulin, glucose regulation indirectly contributes to the prevention and mitigation of DN. Still, polymeric nanoparticles have been used as a delivery system to deliver anti-inflammatory, antioxidant, and anti-fibrotic factors to the kidney along with insulin. This has enhanced renal function, reduced extracellular matrix deposition, and reduced pro-inflammatory cytokines (TNF-α, IL- 6) in preclinical models (178). Kidney-targeted rhein-loaded polyethylenimine polyethyleneglycol-polycaprolactone-co-polyethylenimine (PPP-RH-NPs) nanoparticles were generated to enhance the renal excretion of rhein to treat DN (179). These nanoparticles demonstrated sustained drug release and significant therapeutic outcomes in diabetic models of nephropathy, including reduced serum creatinine concentration and reduced renal fibrosis (179).

#### 6.1.5 Dendrimers

Dendrimers are nanoscale, radially symmetric molecules that possess a well-defined, uniform, and monodisperse structure with an inner shell, an outer shell, and a normally symmetric core (180). Their three traditional types of macromolecular structure are widely recognized to yield highly polydisperse products with diverse molecular masses. All forms of dendrimers have biological properties that include solubility, low cytotoxicity, electrostatic interactions, polyvalency, self-assembly, and chemical stability (180). Another type of nanoparticle with extensive research is the dendrimer-based polyamidoamine (PAMAM) nanoparticle. Considering the fact that it is mainly concentrated in the kidney with an insignificant amount remaining in the other organs, several studies have demonstrated that I-Serine modified PAMAM (G3) dendrimers are highly effective as a proximal tubule-specific drug carrier in the kidney (181, 182). The dendrimer can minimize its adverse effects by effectively directing captopril to the kidney (181, 182).

#### 6.1.6 Micelles

Polymeric micelles are nanoscale carriers consisting of a hydrophilic shell and a hydrophobic core that enhance the solubility and stability of poorly soluble medications. Natural chemicals and antioxidants (such as resveratrol and curcumin) are better transported by micelles in diabetes and its effects (183). They offer tissue targeting, increased release, and reduced toxicity. Due to their high versatility and biocompatibility, micelles remain potential nanocarriers in diabetes therapy despite problems such as variable stability and untimely drug release (183). Recent advances in nanomedicine have provided micelle-based systems as versatile delivery platforms for renal-targeted therapy. A worthwhile advancement is the micelle-hydrogel hybrid method, which offers both local administration and prolonged drug release in kidney tissue. Scientists examined the administration of insulin orally using polymeric nanoparticles and micelles (184). They described a method in which mucoadhesive and permeability-enhancing strategies can be used to enable insulin-carrying micelles to avoid gastric degradation and facilitate absorption in the intestines. These solutions use controlled release, applied together with protective encapsulation, to address the problems of limited bioavailability and enzymatic degradation that afflict oral insulin administration (184). Another study has demonstrated that such an injectable hybrid significantly enhances medication retention and bioavailability and reduces signaling pathways associated with fibrosis, thereby reducing extracellular matrix deposition and scarring of the kidney (185). These findings suggest that micelle-hydrogel hybrids could be a feasible therapeutic approach for treating fibrosis in DN, because fibrogenesis, driven by TGF-beta/Smad signaling and ECM deposition, is an essential stage in the pathogenesis of DN. This system can provide a platform for site-specific, long-acting antifibrotic treatment, integrating the structural integrity of hydrogels with the advantages of micelles (biocompatibility and efficient drug loading) (185).

#### 6.1.7 Nanogels

Nanogels are nanoscale crosslinked polymeric networks with a high capacity to absorb water and medications. They combine the benefits of hydrogels (high loading capacity, hydrophilicity) with nanoscale delivery (excellent tissue penetration, sensitivity to stimuli). They are also capable of stabilizing the fragile treatments (e.g., insulin and peptides) and are often biocompatible and biodegradable (186). They can be loaded with drugs and released on demand due to their structural flexibility, and they can penetrate the tissues because of their small size. Nanogels have also become attractive as a delivery vehicle to insulin, antioxidants, and anti-fibrotic agents because of their capability to address the metabolic dysfunction, oxidative stress, and inflammation -the major hallmarks of the DN development (186). The most-studied application of nanogels is insulin delivery. Low absorption and breakdown of the gastrointestinal tract interfere with the oral administration of insulin. Insulin can be entrapped in nanogel formulations that protect it from enzymatic degradation and release it at a controlled rate in response to physiological stimuli. Self-assembled insulin nanogel complexes are more stable, pH-responsive, and absorbable in diabetic rat models, resulting in improved glycemic control compared to free insulin (184). Indirectly, these platforms help reduce the formation and progression of DN by improving systemic glucose control. Other nanogel-based systems are also explored as antifibrotic treatment and renal protection. Pathways such as TGF-2/Smad signaling lead to extracellular matrix deposition and glomerular scarring in DN, which nanogels can inhibit by delivering drugs locally and sustainably (185). Injectable nanogel-hybrid systems have also been shown to be effective in reducing pathological remodeling and inhibition of renal fibrosis in preclinical models. This ensures that nanogels can be used as an antifibrotic surface in the treatment of DN (185).

### 6.2 Advantages of nanocarriers

**Figure 5:**
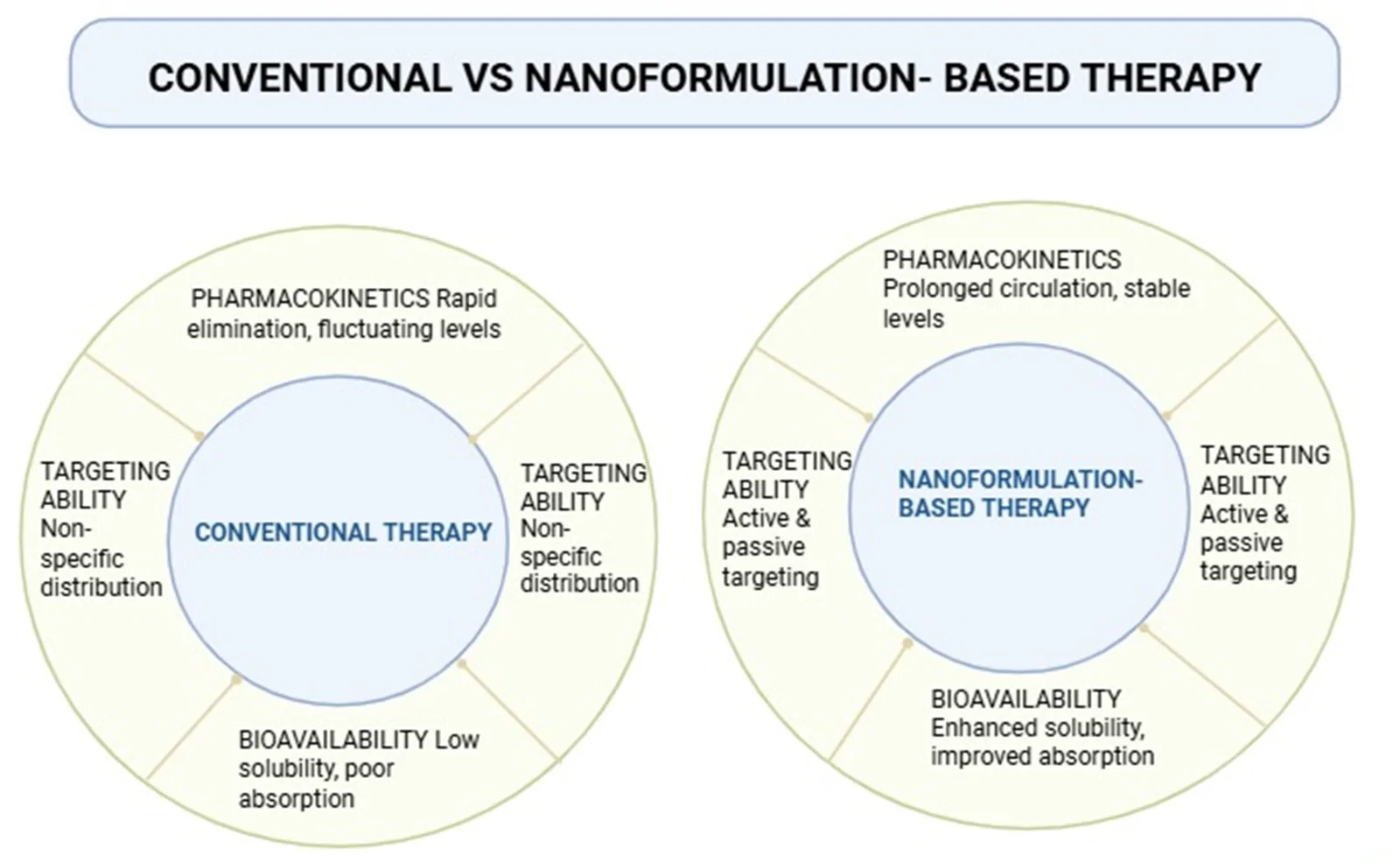
Comparative overview: conventional vs. nanoformulation-based therapy.

Nanocarriers also have great benefits over the conventional mode of drug delivery, such as better solubility, stability, and controlled release of the drug, as shown in (Figure 5). All these characteristics facilitate more effective administration of drugs to particular tissues or cells and minimize systemic toxicity and increase treatment efficacy (187).

#### 6.2.1 Improved drug solubility and stability

Nanocarriers have several advantages compared to traditional drug delivery methods, including increased drug solubility, stability, and release control. These properties reduce systemic toxicity and enhance therapeutic efficacy by improving the delivery of medication to specific tissues or cells (188). The solubility and stability of hydrophobic medications in the body's aqueous environment are enhanced when they are encapsulated in nanocarriers, and they are also protected against degradation in the gastrointestinal tract (188, 189). As an example, lutein-charged zein-derived peptide nanoparticles were found to be significantly more soluble and more stable in simulated stomach and intestinal fluids than free lutein (189). Secondly, inclusion of drugs in nanocarriers may result in increased wettability, aggregation prevention, and amorphization, conversion of crystalline frameworks to amorphous ones that are more soluble (188). This is necessary when medications are not highly soluble under varying physiological conditions, which often leads to reduced absorption rates and other therapeutic effects. Improved solubility within the therapeutic window of plasma drug levels reduces adverse effects and enhances efficacy, leading to more predictable therapeutic and pharmacokinetic responses (188).

#### 6.2.2 Enhanced bioavailability and targeting

Because nanocarriers can deliver drugs to specific cells or tissues, they are highly beneficial for improving drug bioavailability and targeting. Such a targeted approach increases the therapeutic index of drugs and reduces adverse effects on systems. Nanoparticles may also be functionalized to respond to specific receptors on target cells, ensuring that the drug is delivered to the areas of need (187, 188). This particular distribution is highly essential when treating diseases in which conventional medicine would damage the normal cells. Magnetic nanoparticles (MNPs) can be engineered to exhibit improved ability to cross biological membranes and deliver drugs in a controlled manner, enhancing bioavailability and yielding more dependable treatment effects across diverse patient populations (188). External magnetic fields can also be used to deliver MNPs to specific locations, including inflammatory or tumor sites (188). Specific drug delivery systems, particularly nanocarrier-based systems, are more effective than free drug delivery because they reduce systemic toxicity and enhance absorption, diffusivity, and overall therapeutic responses (190).

#### 6.2.3 Controlled and sustained release profile

Nanocarriers aim to achieve the regulated and sustained release of therapeutic compounds, offering significant advantages in maintaining medication levels within the therapeutic range over extended periods (187, 191). This increases patient adherence and reduces the dosage rate. As an example, polymeric nanoparticles can be programmed to deliver drugs in a controlled manner, which ensures therapeutic levels are sustained. Doxorubicin has exhibited evident sustained release and high encapsulation efficiency in magnetic conducting polymer/mesoporous SiO2 yolk/shell nanostructures (188, 192). One of the major factors that determines the toxicity and treatment efficacy of nanoparticles *in vivo* is their ability to control release kinetics. This control can be achieved through several engineering strategies, including the production of cross-linkable lipid shell nanoparticles, which can be used to modify the kinetics of drug release without altering other nanoparticle characteristics (193). Moreover, it has been shown that stimuli-responsive nanocarriers can be used to control drug release patterns via triggered release. Amino-starch nanoparticles were used as an example of their potential as a controlled-release nanocarrier and were found to release curcumin gradually after 12 h (194).

#### 6.2.4 Reduced off toxicity

Nanocarriers will play a central role in reducing off-target toxicity by targeting sick tissues or cells and reducing contact with healthy ones (195). Targeted delivery can be obtained either by passive targeting (enhanced permeability and retention effect) or active targeting by ligand-receptor interactions, which may allow for more focused accumulation of nanocarriers at diseased tissue or at specific cells and less exposure to healthy tissues (196). This specificity is necessary for drugs with a narrow therapeutic index or for those with severe systemic effects (188). Furthermore, cellular uptake at the therapeutic site while minimizing distribution to non-target tissues is considered an important strategy for reducing systemic toxicity (197). It has been demonstrated that the *in vivo* toxicity of angiogenesis inhibitors can be significantly reduced when they are encapsulated within polymeric nanoparticles (198). The use of externally stimuli-sensitive nanocarriers has the advantage of maximizing drug delivery to the target site and minimizing side effects. Stimuli-responsive systems can trigger drug release in response to disease-associated endogenous stimuli, such as pH, redox conditions, enzymes, or hypoxia, or externally applied stimuli, thereby providing more spatially and temporally controlled drug release at the target site (199). Nanoparticles could reduce the toxicity of therapeutic cargo, as formulations of established hepatotoxic compounds in nanoparticle form have been shown to be associated with reduced hepatotoxicity compared to their small-molecule analogs (187). Therefore, nanocarrier-mediated targeting may improve the therapeutic index of potent drugs by increasing drug exposure at the desired site while reducing unnecessary exposure to healthy tissues (200).

#### 6.2.5 Mechanisms of renal targeting

**Figure 6:**
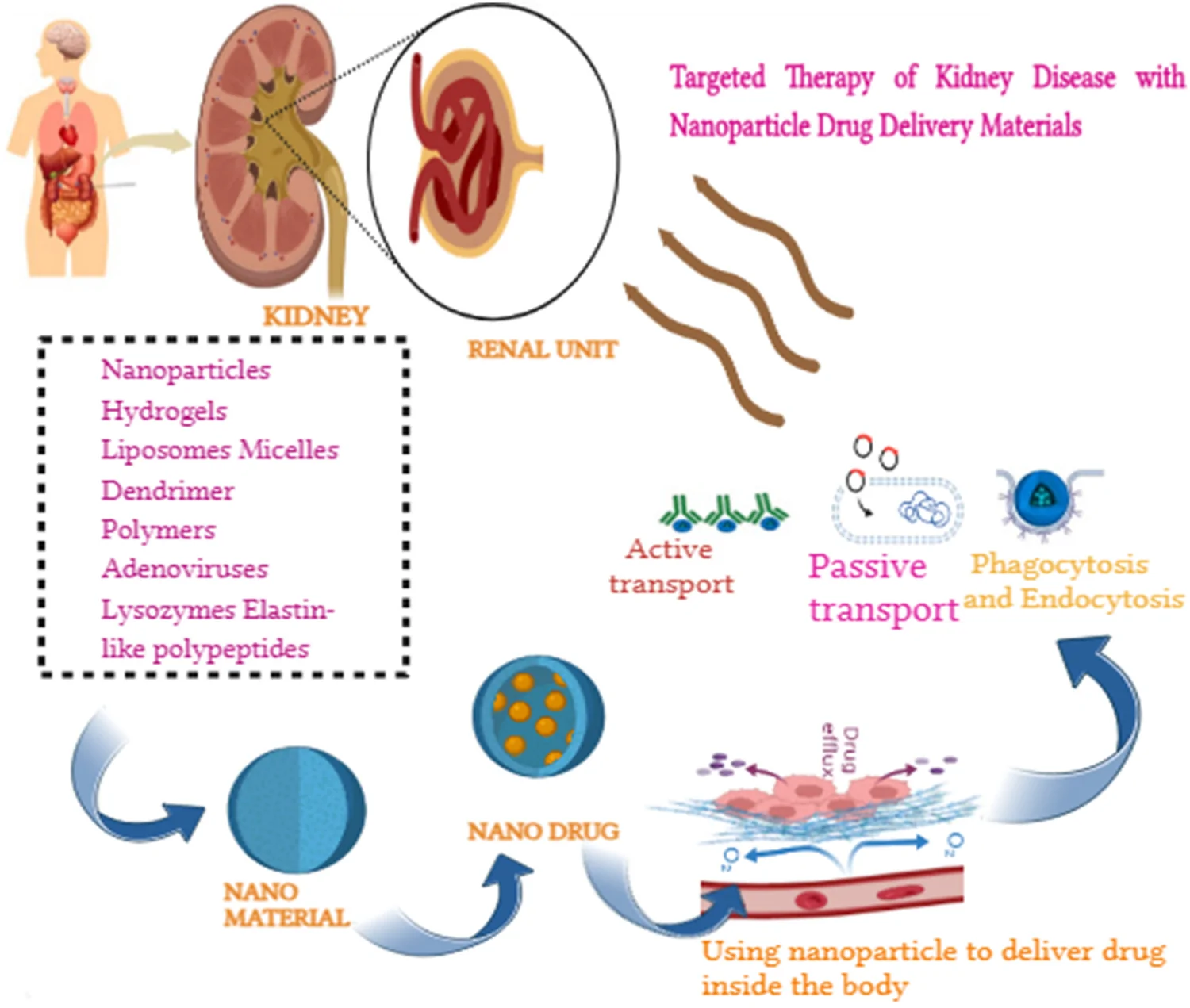
Schematic representation illustrating the mechanism of renal drug targeting using nanocarrier-based delivery systems.

Efficient renal targeting is crucial for treating DN, and nanocarriers employ both passive and active mechanisms to achieve this specificity, as shown in (Figure 6). These mechanisms rely on various factors such as particle size, charge, and the use of specific ligands (188).

#### 6.2.6 Passive vs. active targeting

In renal therapies, kidney-specific nanocarriers reduce off-target effects and enhance therapeutic efficacy through passive and active targeting mechanisms (189). With factors such as particle size, charge, shape, and material properties contributing to organ specificity, passive targeting takes advantage of the kidney's distinct physiological features (188, 189). Increased vascular permeability and localized inflammation in the glomerulus, for instance, increase the likelihood that nanoparticles would passively target and aggregate there in the nephritic state. This phenomenon is known as the enhanced permeability and retention (EPR) effect. This effect constitutes passive targeting, especially when paired with nanocarriers designed for prolonged circulation in the bloodstream.

Active targeting, on the other hand, uses ligand-receptor interactions to achieve renal specificity. To transfer chemicals, peptides, or antibodies to target cells via receptors, nanocarriers must be modified (18, 188). The selective transport of nano-formulations to specific locations has been enabled by a variety of targeting ligands, including antibodies and non-antibody ligands (e.g., transferrin, RGD, folic acid). Adapting to receptor peptide sequences facilitates active targeting. Overall, both passive and active renal targeting are complementary methods to enhance renal drug delivery. Passive targeting is relatively easy to achieve and primarily depends on physicochemical and physiological properties, but it may not be as cell-specific as active targeting and may result in variable renal accumulation. Active targeting, conversely, may offer improved cellular and site specificity through ligand-receptor interactions, but to be effective, receptors must be present, and additional potential problems may arise with ligand stability, immunogenicity, manufacturing, and regulatory considerations.

#### 6.2.7 Intra-renal site-specific targeting

Site-specific delivery to different compartments of the kidneys is also gaining attention as a key approach to enhance the therapeutic efficacy and safety of nanomedicines. The kidney is a structurally and functionally complex organ in which the glomerulus, podocytes, tubular epithelium, and mesangial cells contribute to filtration, barrier maintenance, solute reabsorption, and inflammatory and fibrotic processes (201). Thus, if the nanocarrier does not reach the site of disease activity, nonspecific renal accumulation may not confer therapeutic benefit. Therefore, nanocarrier-based approaches have been examined to selectively target distinct intra-renal sites, such as the glomerular capillary network, podocytes, tubular epithelial cells, and mesangial cells (202). For diseases with glomerular injury, targeting the glomerular compartment with nanoparticles that interact with glomeruli components or that accumulate in that compartment is of particular interest. Targeting podocyte-specific therapies may offer additional therapeutic advantages in diseases involving podocyte injury or dysfunction, including proteinuric kidney diseases. In addition, the interest of tubular targeting is that the tubular epithelial cells play a major role in drug-induced injury, inflammation, oxidative stress, and fibrosis. The involvement of mesangial cells is of special interest in diseases associated with mesangial proliferation, extracellular matrix accumulation, and glomerulosclerosis (203). The success of intra-renal targeting relies on the balance between the biological and the physicochemical properties of the target site and the nanocarrier, respectively. The effects of particle size, surface charge, morphology, surface chemistry, biodegradability, and ligand density on circulation, renal filtration, tissue penetration, cellular uptake, and intracellular trafficking can be influenced. Moreover, disease-associated changes in renal vascular permeability, extracellular matrix composition, receptor expression, and cellular phenotype can significantly affect nanocarrier distribution (204). Thus, optimal targeting of the nanocarriers to the renal compartment requires careful optimization of nanocarrier properties based on the targeted renal compartment and disease status, along with the selection of an appropriate targeting ligand. These site-specific strategies have the potential to increase drug concentration in the target tissue and minimize exposure to non-target renal and systemic tissues, but they require reproducible targeting efficiency, safety, and scalable manufacturing to be implemented in the clinic (204). Such site-specific approaches may enhance local drug concentrations while reducing exposure to non-target renal and systemic tissues, although their clinical translation remains dependent on reproducible targeting efficiency, safety, and scalable manufacturing.

#### 6.2.8 Size- and charge-dependent kidney accumulation

One of the most important factors influencing the accumulation of nanoparticles in the kidney is their size and charge. Although size and surface charge are significant factors in renal clearance and accumulation, they cannot be used as absolute or universally applicable limits for predicting renal fate. The combined effects of hydrodynamic diameter, particle geometry, deformability, surface charge, surface chemistry, protein corona formation, and interactions with the GFB govern renal biodistribution. Significantly, the surface characteristics and size of nanoparticles can vary after exposure to biological fluids due to protein absorption and the formation of a corona (205). Furthermore, renal disease can negatively impact the structure and function of the GFB, including changes in endothelial permeability and GBM properties, as well as alterations in podocyte architecture, which can affect nanoparticle filtration and retention. This may suggest that there is no one-size-fits-all criterion for defining size- or charge-dependent renal behavior, and that it should be interpreted in light of the nanoparticles’ design and the physiology/pathology of the kidney (206). Efficient renal clearance and urinary elimination occur for very small nanoparticles, but clearance depends on other factors. The hydrodynamic diameter (geometry, deformability, surface properties, and integrity of the GFB) all contribute to whether the nanoparticles are filtered, retained in the GFB, or later taken up by renal cells. Therefore, the reported size range should not be interpreted as absolute limits of renal clearance (158).

Positively charged nanoparticles are rapidly cleared from the kidney due to their greater capacity to bind proteins and engage the mononuclear phagocytic system. Macrophages prefer to absorb negatively charged nanoparticles over neutrally charged ones (207). Charges of ±15 mV or less make nanoparticles less likely to be absorbed by macrophages and improve blood circulation. Endosomal escape is possible for cationic nanoparticles, which readily cross cellular membranes (208). Because the glomerular basement membrane is anionic, it readily adsorbs positively charged nanoparticles rather than neutrally charged ones. It has been discovered that ultrasmall nanoparticles (less than 5 nm) with a negative surface charge may penetrate glomerular capillaries and gradually accumulate in mesangial cells for up to 30 days. This suggests that charge plays a major role in renal absorption of extremely small nanoparticles. Surface charge can have significant effects on protein adsorption, interactions with biological membranes, uptake by mononuclear phagocytes, and renal transport, but these effects are highly dependent on the composition and surface chemistry of a nanoparticle, the formation of a protein corona, and the biological environment. Thus, a general ±15 mV rule for macrophage uptake or renal biodistribution of various nanocarriers cannot be drawn (207, 208).

#### 6.2.9 Use of ligands (e.g., peptides, antibodies) for glomerular/podocyte targeting

A key benefit of nano-platforms is loop conjugation, which facilitates site-specific delivery and retention. By adding different biological ligands to nanoparticles, selectivity and efficiency can be increased, while systemic accumulation and potential toxicities can be avoided. To actively target specific receptors on target cells, such as those on glomeruli or podocytes, ligands, such as peptides and antibodies, can be bound to the surface of the nanocarrier (209). For instance, rhein-loaded liponanoparticles with lipid layers altered by a kidney-targeted peptide (KTP) enhanced renal cellular uptake and endocytosis through a non-lysosomal mechanism (210). Collagen IV, αvβ3 integrin receptors, megalin receptors, and angiotensin II receptors have shown promise as potential targets for medication delivery to treat DN. But more accumulation in renal tissues does not guarantee therapeutic efficacy in site-specific delivery (211). The renal targeting efficiency needs to be taken into account, including the fraction of the injected dose reaching the kidney, kidney-to-liver and kidney-to-spleen distribution ratios, intra-renal and subcellular localization, and renal retention and clearance kinetics (212). It is important that targeted nanocarriers exhibit superior therapeutic efficacy to their non-targeted counterparts, and that therapeutic efficacy is not confounded by the nanocarrier's intrinsic biological effects but rather by the encapsulated therapeutic payload (213). These parameters provide a better idea of the extent to which renal accumulation is improved and whether this is an effective and clinically relevant form of renal targeting.

## 7 Nanoformulation-based therapies in DN: preclinical and clinical studies

**Figure 7:**
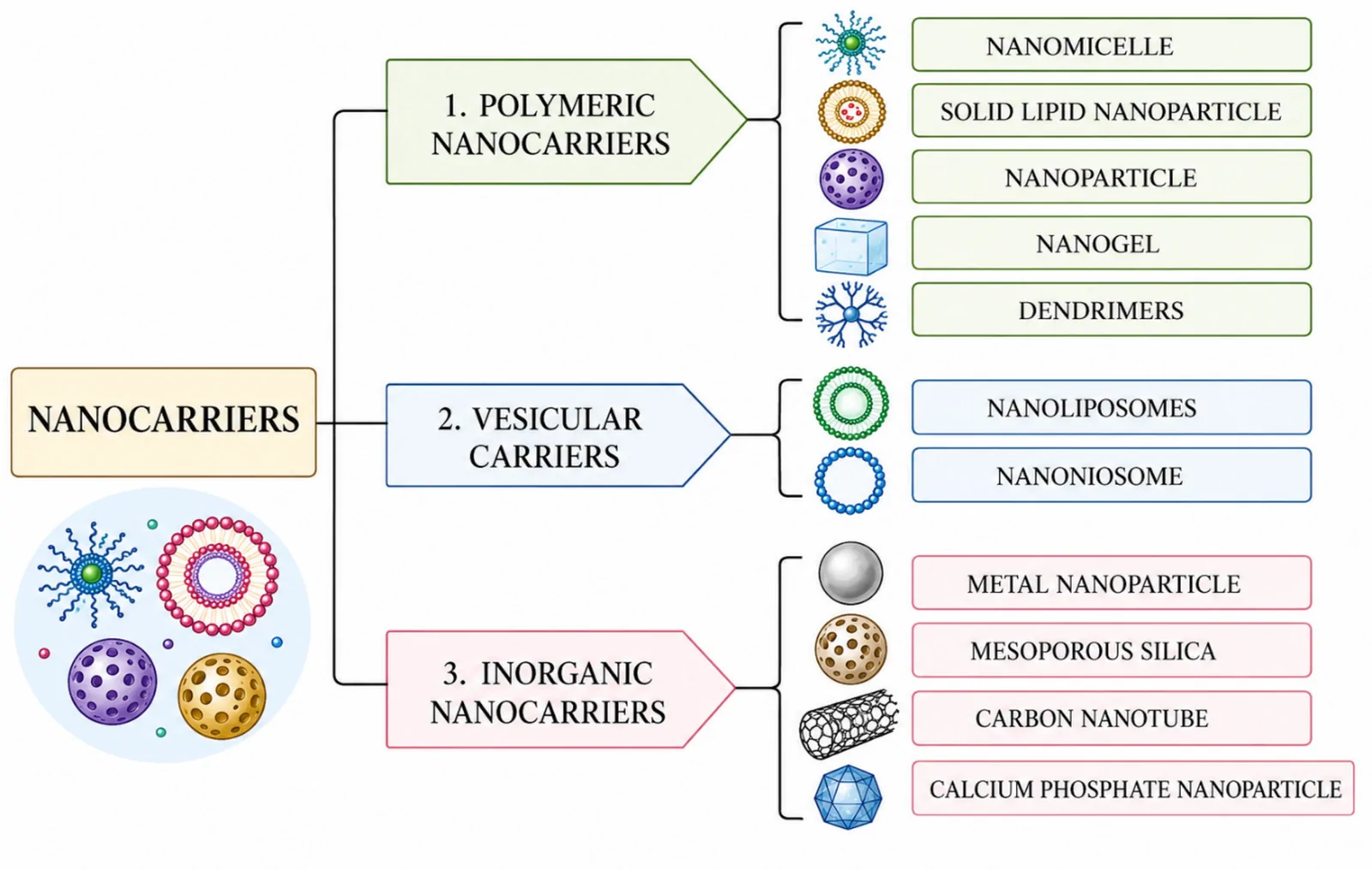
Types of nanocarriers used in DN therapy.

Nanoformulations have attracted increasing attention for therapeutic applications because of their ability to cross biological barriers and enhance drug bioavailability. Antidiabetic drugs can be dispersed, implanted, encapsulated, or incorporated into nanoparticles via a nanoplatform for drug delivery, with advances in biochemistry and nanotechnology (159). Nano formulations, including liposomes, polymeric nanoparticles, solid lipid nanoparticles, and nanomicelles, have been shown in preclinical research (Figure 7) to enhance antioxidant and anti-inflammatory activities, reduce fibrosis, and improve renal drug delivery, as shown in Table 3. These approaches have been explored with agents such as curcumin, resveratrol, insulin, and siRNA, which have shown improved renal outcomes in animal models of DN (214). Although translation is still limited in clinical settings, few trials assess antioxidant and antidiabetic drugs based on nanocarriers. Compared with traditional formulations, early findings suggest improved glycemic and renal outcomes, fewer side effects, and enhanced pharmacokinetics (159).

**Table 3:** Summary of preclinical experimental studies evaluating nanoformulation-based drug delivery systems in DN models.

Study	Nanoformulation	Animal model	Outcome	Ref.
Antioxidant	Myricitrin-loaded SLNs (solid lipid NPs)	STZ-NA diabetic mice	Decreased oxidative stress, decreased apoptosis, improved histology vs. free myricitrin.	(174)
Antioxidant	Rutin + SeNPs	STZ diabetic rats	Upregulation of Nrf2/HO-1 reduced oxidative & inflammatory markers; renoprotection.	(215)
Antioxidant	Selenium nanoparticles	STZ diabetic rats	Attenuated oxidative stress markers in diabetic models.	(216)
Antidiabetic/Metabolic-modulating	Nanocurcumin (PLGA/ nanoparticulate curcumin) ± insulin	STZ diabetic rats	Reduced albuminuria, improved glycemia, and reduced glomerular apoptosis.	(217)
Antidiabetic/Metabolic-modulating	Selenium nanoparticles (SeNPs)	STZ diabetic rats	Anti-hyperglycemic effects, improved renal biochemical markers.	(218)
Antidiabetic/Metabolic-modulating	AuNPs + dapagliflozin	STZ diabetic rats	Synergistic antidiabetic/renoprotective effects; reduced fibrosis & apoptosis via miR-192/miR-21 modulation.	(219)
Anti-inflammatory	Kidney-targeted rhein-loaded liponanoparticles	STZ diabetic rats	Preferential renal accumulation; reduced proteinuria and renal inflammation/histologic injury.	(210)
Anti-inflammatory	Adropin in ROS-responsive nanocapsules	DN rodent models/HK-2 cells	ROS-triggered release → reduced renal lipid toxicity & inflammation, improved endothelial function.	(220)
Antifibrotic	Gold nanoparticles	STZ diabetic rats	Reduced albuminuria, inhibited podocyte injury; signals of reduced fibrosis.	(221)
Antifibrotic	AuNPs + dapagliflozin	STZ diabetic rats	Reduced Fibrosis markers and improved histology via miRNA pathways.	(219)
Anti-apoptotic	Nanocurcumin (PLGA nCUR)	STZ diabetic rats	Reduced glomerular apoptosis and pro-apoptotic signaling (P38/P53 axis).	(217)
Anti-apoptotic	Myricitrin-SLNs	STZ-NA diabetic mice	Anti-apoptotic effects in renal tissue; preserved histology.	(174)

### 7.1 Overview of nano-formulated therapeutic agents investigated in DN

Various nanoformulated medicines have been investigated to enhance renal targeting, bioavailability, and therapeutic efficacy while reducing systemic side effects in preclinical models of DN, as shown in Table 4. The tacrolimus-loaded albumin nanoparticles functionalized with RGD peptides showed targeted podocyte delivery, podocyte integrity renewal, and a substantial decrease in proteinuria in diabetic rats, addressing the toxicity of calcineurin via site-specific action (222). Carvedilol in a self-nanoemulsifying drug delivery system enhanced solubility and renal accumulation, leading to strong antioxidant and antifibrotic activity, as compared to free drug, glomerular and tumoral damage decreased (223). Curcumin nano-formulations, such as liposomal carriers and polymeric nanoparticles, demonstrated reno-protective effects by reducing oxidative stress and inflammatory signaling, providing a natural antioxidant strategy. In the same way, resveratrol-loaded nanoparticles in DN models provided enhanced stability and extended circulation duration, along with suppressing renal fibrosis and oxidative injury (224). Moreover, another study, like dapagliflozin- encapsulated nanocarriers, has been studied for enhanced renal absorption, glucose-lowering efficacy, along with additional protection against renal inflammation (225). In DN, all these studies emphasize that nano-formulation techniques enhance therapeutic index, renal targeting, and disease-modifying potential, whether by delivering genetic payloads or repurposing existing medications. However, the translation to clinical studies is still limited.

**Table 4:** Comparative overview of nanocarrier-based drug delivery platforms investigated in DN research.

S. No	Nanocarrier type	Composition	Drug/Compound	Target	Key advantages	Ref
1	Metallic nanoparticles	Selenium nanoparticles	SeNPs	Kidney	Strong antioxidative effect; improve markers such as urea, creatinine; mitigate apoptosis; can be combined with other agents for synergism	(226)
2	Metallic nanoparticles	ZnO nanoparticles	ZnO	Podocytes, kidney	Inhibition of fibrosis, oxidative stress, inflammation; protection of renal function; improve BUN, creatinine, urinary albumin etc.	(226)
3	Polymeric nanoparticles	PLGA NPs	Curcumin; Phe-Tyr peptide; rhein	Kidney	Improve bioavailability; reduce required dose; mitigate glomerular apoptosis, reduce proteinuria, improve histopathology vs. free drug	(160)
4	Lipid-based nanocarriers- Solid Lipid Nanoparticles	Lipid core, surfactants	Myricitrin	Kidney	Better absorption, longer release, reduced oxidative damage vs. free myricitrin; lower toxicity; better cell uptake	(160)
5	Phospholipid nanoparticles	celastrol loaded in phospholipid nanoparticles, coated with a VCAM-1 targeting peptide	Celastrol	Glomerular endothelial cells & podocytes	Active targeting reduces off-target toxicity; improved delivery to glomerular cells; more potent anti-inflammatory effects; preserved glomerular structure	(160)
6	Lipid-polymer hybrids	Polymeric core + lipid shell + targeting modifications	Rhein	Renal tubular cells, mesangial cells, endothelial cells, podocytes	Better renal cell uptake; improved therapeutic efficacy vs. free drug or non-modified NP; sustained release; targeted distribution	(160)
7	Inorganic nanocarriers – Silver NPs (AgNPs)	AgNPs, often loaded with bioactive plant extract	*Momordica charantia* leaf extract	Kidney	Enhanced PPARα/*γ*, normalized kidney injury molecules, reversed pro-inflammatory/profibrotic gene changes vs. free extract	(160)
8	Exosomes	ADSC-derived exosomes modified with miR-204	miR-204	Target METTL7A/CIDEC m6A methylation → mitochondrial dysfunction & oxidative stress in DN	In DN rats, these exosomes reduced injury, oxidative stress, improved mitochondrial function and reduced apoptosis	(227)
9	Cyclodextrin polycationic NPs	Polycationic cyclodextrin nanoparticle complex	siRNA (glomerular targets)	Glomerular mesangium	Preferential accumulation in glomerular mesangium after IV dosing -promising for siRNA therapeutics in DN.	(228)
10	Dendrimers	PAMAM dendrimers	Small antioxidants, nucleic acids, NO donors	Oxidative stress, targeted delivery to kidney cells	High loading and tunable surface chemistry for targeting; size/charge can be tuned for renal localization	(229)

### 7.2 Natural compounds (e.g., curcumin, resveratrol) in nanoform

Natural bioactive molecules, including curcumin and resveratrol, have shown potential renoprotective effects against DN due to their potent antioxidant, anti-inflammatory, and antifibrotic activities. However, their therapeutic potential is constrained by limited aqueous solubility, lowered oral bioavailability, and fast metabolism. Nanotechnology-based drug delivery systems were developed to overcome these barriers. Various curcumin nanoforms have been shown to improve systemic bioavailability and prolong drug release. In preclinical models, nanoforms of curcumin showed significantly reduced albuminuria, tubulointerstitial fibrosis, and glomerulosclerosis by suppressing activation of TGF-β/Smad and NF-κB. Liposomes, polymer carriers, and curcumin nanoparticles were shown to prolong drug release, and improve systemic bioavailability (214).

Preclinical research showed that inhibiting TGF-β/Smad signaling and NF-κB activation with nano-curcumin dramatically reduced tubulointerstitial fibrosis, glomerulosclerosis, and albuminuria. In the same way, resveratrol nanoformulation enhanced chemical stability and pharmacokinetics, leading to a more marked reduction in oxidative stress and kidney fibrosis than free resveratrol in diabetic mouse models (230).

These nanoformulations enhanced podocyte survival, maintained renal function, and decreased apoptosis markers and extracellular matrix accumulation. Collectively, the application of nanotechnology has revived the therapeutic potential of curcumin and resveratrol for DN, providing renal targeting and disease-modifying efficacy, though translation into clinical trials is still awaited.

### 7.3 Nanoparticles carrying anti-fibrotic/anti-inflammatory agents

In recent years, the use of nano-formulations of antifibrotic and anti-inflammatory drugs has increased because renal fibrosis and chronic inflammation are primary causes of DN. Nanocarriers offer enhanced solubility, prolonged circulation time, and improved renal targeting, thereby intensifying therapeutic efficacy while reducing systemic side effects. For example, tacrolimus-loaded albumin nanoparticles functionalized with RGD peptides achieved selective podocyte uptake, significantly reducing proteinuria and glomerular injury while minimizing nephrotoxicity (222). Similarly, in a diabetic animal model, siRNA-loaded polymeric nanoparticles targeting inflammatory pathways such as NF-κB and pro-fibrotic mediators, including TGF-β1, effectively reduced cytokinin development and kidney fibrosis. Lipid-based nanocarriers carrying antifibrotic medicine pirfenidone exhibited a greater reduction in extracellular matrix deposition when compared to free medicine (224). Collectively, these findings suggest that nanoparticle-based platforms can potentiate anti-fibrotic and anti-inflammatory therapies, representing a promising approach to slow DN progression. However, clinical translation remains limited, and further research is required to optimize safety, scalability, and targeted delivery.

### 7.4 Clinical trial progress and regulatory status

In the last decade, the treatment paradigm for DN has shifted from glucose-focused care to treatments that directly retard kidney disease progression, as evidenced by clinical trial success and regulatory approvals. Large, randomized outcome trials of sodium-glucose cotransporter-2 inhibitors (SGLT2i), specifically CREDENCE, DAPA-CKD, and EMPA-KIDNEY, showed uniform renal benefit (less progression to kidney failure and slower decline in eGFR) and have positioned SGLT2i as a treatment standard for CKD with and without diabetes (231).

Regulatory agencies have acted on this evidence: SGLT2 inhibitors are now universally recommended in CKD guidelines, and other new agents have received formal approval. The non-steroidal mineralocorticoid receptor blocker Finerenone was approved by the FDA and EMA for CKD with type 2 diabetes following the FIDELIO-DN/FIGARO-DN program, demonstrating reductions in kidney and cardiovascular outcomes; Finerenone is an important, guideline-recommended adjunct to blockade of the renin–angiotensin system and SGLT2i in suitable patients (113, 232).

The current trial pipeline is diverse and increasingly focused on combination strategies and precision targets. Several ongoing and recently registered trials are testing GLP-1 receptor agonists (e.g., semaglutide) for renal endpoints, selective anti-inflammatory agents, anti-fibrotic candidates, and novel biologics; early-phase programs (including INV-202 and other mechanistic agents) are exploring safety, pharmacokinetics, and biomarker effects in DN populations. These trials aim to move beyond surrogate metabolic benefits and demonstrate meaningful reductions in albuminuria and hard kidney outcomes (233).

In contrast, nanomedicine and nanoparticle-based therapeutics for DN are mostly in their preclinical and translational phases. A number of targeted nanoparticles (drug- and siRNA-loaded platforms) have been shown to reduce inflammation, fibrosis, and proteinuria in animal DN models, and several reviews have been published on the theranostic potential of such platforms; however, clinical trials with renal-targeted nanotherapeutics in DN are rare to date and to date, no nano-formulation has been broadly approved by the regulators for DN treatment. The translational barriers encompass extensive toxicity/safety testing, renal clearance predictivity, process scalability for manufacturing, and pre-clinical human studies, which require strong renal imaging endpoints and renal biomarkers (159). The following trends are noteworthy for DN therapeutics: (1) the trend of using hard renal endpoints (sustained eGFR decline, ESKD), or composite endpoints that include a renal component in addition to the primary clinical endpoint; (2) platform trials and combination trials testing additive effects of agents (e.g., SGLT2i + Finerenone); (3) using more biomarkers and imaging to provide early proof-of-mechanism evidence in a more enriched CKD population; and (4) focus on safety signals (hyperkalemia with MRA, volume status with SGLT2i, off-target effects with novel biologics). There are a couple of expert reviews that conclude that there is a need for clinical benefit on hard outcomes and compelling safety/PK data in CKD populations for the next generation of approvals (234). Class changing agents (CAs) (SGLT2 inhibitors, finerenone) have been proven to be effective in clinical trials and are now recommended in standard clinical care and approved in the labels for T2D-associated CKD. The late-stage pipeline features combinations and agents targeting inflammation/fibrosis, with nanomedicine making its way into pre-clinical programs but not yet having a robust clinical program or approvals. The next steps will rely on the careful execution of early phase human studies, on defining common metrics and on addressing manufacturing and safety concerns for novel platforms in a translational manner.

## 8 Limitations and future directions

Although nanotechnology-based drug delivery systems have shown significant promise in preclinical models of DN, translation into clinical practice remains challenging. Several scientific, regulatory, and translational barriers need to be addressed before nano-formulations can be widely adopted for DN management (159).

### 8.1 Challenges in scalability and regulatory approval

The successful application of nanocarriers in DN is hindered by manufacturing and regulatory hurdles. While small-scale laboratory synthesis can produce uniform, stable nanoparticles, scaling up to industrial production often results in batch-to-batch variability in particle size, surface charge, and drug-loading efficiency (168). Regulatory agencies such as the FDA and EMA require rigorous standardization, stability studies, and detailed pharmacokinetic profiles before approval. Moreover, the absence of universally accepted guidelines for nanomedicines adds uncertainty to the approval pathway, making translation more complex compared to conventional drugs (168).

### 8.2 Long-term toxicity and biocompatibility issues

Despite encouraging short-term safety profiles, the long-term toxicity and biocompatibility of many nanocarriers remain poorly understood. Nanoparticles may accumulate in off-target organs such as the liver, spleen, and lungs, raising concerns about chronic toxicity and immune responses (235). For example, cationic polymeric nanoparticles may induce cytotoxicity, while certain inorganic nanoparticles carry risks of oxidative stress and genotoxicity. Furthermore, renal pathology itself may alter nanoparticle clearance, complicating toxicity predictions. Comprehensive *in vivo* studies and long-term safety assessments are needed to ensure biocompatibility, particularly in patients requiring lifelong therapy (236, 237).

### 8.3 Need for kidney-specific nanocarriers

Although nanotechnology improves drug solubility and targeting, most current formulations lack renal specificity. Passive targeting strategies relying on size and charge often result in suboptimal accumulation within the kidney (238). Active targeting using ligands such as peptides, antibodies, or aptamers holds promise but requires precise optimization to recognize glomerular, podocyte, or tubular markers without affecting normal tissue function. The development of kidney-specific nanocarriers with high affinity, stability, and minimal immunogenicity will be critical for enhancing therapeutic efficacy in DN (239).

### 8.4 Personalized nanomedicine approaches

DN is a heterogeneous disease, with variations in genetic predisposition, disease stage, comorbidities, and response to therapy. A “one-size-fits-all” nanoformulation may therefore be insufficient (240). Advances in precision medicine, omics technologies (genomics, proteomics, metabolomics), and biomarker discovery can facilitate personalized nanomedicine strategies, enabling patient-specific selection of nanocarrier type, drug payload, and dosing regimen (241). Moreover, integrating theranostic nanoparticles may allow simultaneous diagnosis and monitoring of therapeutic response, paving the way for individualized treatment plans.

## 9 Conclusion

Though much has been accomplished in diabetes care, the prevalence of CKD and ESRD continues to be a significant public health problem and contributor to the burden of disease in the United States. It is the result of chronic hyperglycemia, oxidative stress, chronic inflammation, and activation of profibrotic pathways, ultimately leading to damage to podocytes, increased BM thickness, and tubulointerstitial fibrosis. From a clinical perspective, diagnosis and staging remain the main pillars of albuminuria and eGFR, with new biomarkers and advanced imaging techniques emerging. However, a significant amount of structural damage to the kidneys has already occurred when abnormalities in these traditional markers are first diagnosed. Examples of the ongoing disconnect between biological injury and clinical detection include normoalbuminuric DN, variability in eGFR estimation, and the invasive nature of renal biopsy. Treatment with intensive glycemic control, RAAS blockade, SGLT2 inhibitors, GLP-1 receptor agonists, lipid-modifying agents, and lifestyle interventions is clearly beneficial for renal and cardiovascular outcomes. Still, they do not often reverse the damage that has already taken place. They are often limited by suboptimal bioavailability, rapid clearance, off-target toxicity, and complex dosing regimens that are difficult to adhere to over the long term. Kidney function decreases, and dose adjustments become more challenging, as do the risks for adverse events. These restrictions make it clear that strategies that deliver renoprotective agents directly to the kidney and spare other organs are important. Nanotechnology provides a unified solution to many of these needs and wants. Liposomes, solid lipid nanoparticles, polymeric nanoparticles, dendrimers, micelles, and nanogels can increase the solubility and stability of poorly soluble drugs, allow for controlled and sustained release of the active via size, charge, and ligand-mediated targeting. Preclinical data have shown that encapsulating antioxidants, anti-inflammatory and antifibrotic agents, and antidiabetic drugs yields significant benefits: decreased proteinuria, inhibited oxidative stress, suppressed inflammatory pathways, and fibrosis prevention. The use of natural ingredients formulated at the nanometric scale, such as curcumin and resveratrol, is reviving the study of these molecules, as they can overcome certain pharmacokinetic limitations and enhance their renoprotective properties. But it is not a simple road to go from bench to bedside. Technically, it is challenging to scale up nanoparticle production while maintaining controlled size, surface properties, and drug loading. Long-term biodistribution and toxicity, particularly in the liver, spleen, and lungs, are not fully understood, and clearance and tissue exposure are changed with CKD. Regulatory considerations for nanomedicines are still being developed, and specific physicochemical characterization, comprehensive safety information, and carefully designed clinical trials, clearly outfitted with renal endpoints, are needed. The field requires the development of kidney-targeting nanocarriers that can efficiently deliver therapeutics to glomerular, podocyte, or tubular compartments, with minimal future immunogenicity. Fusing omics-based profiling with new biomarkers could enable the development of personalized nanomedicine, in which the type of nanocarrier, payload, and dosage would be optimized for a patient's disease stage and risk profile. Theranostic platforms, in which imaging and therapy are integrated into a single nano-platform, can enable real-time tracking of renal drug delivery and therapeutic response.
